# Epac2 Deficiency Compromises Adaptation to Dietary Acidification by Decreasing H^+^ Transport in the Renal Nephron

**DOI:** 10.1093/function/zqaf048

**Published:** 2025-10-14

**Authors:** Kyrylo Pyrshev, Anna Atamanchuk, Wenli Yang, Mariya Kordysh, Fang Mei, Oleg Zaika, Xiaodong Cheng, Oleh Pochynyuk

**Affiliations:** Department of Integrative Biology and Pharmacology, The University of Texas Health Science Center at Houston, Houston, TX 77030, USA; Department of Integrative Biology and Pharmacology, The University of Texas Health Science Center at Houston, Houston, TX 77030, USA; Department of Integrative Biology and Pharmacology, The University of Texas Health Science Center at Houston, Houston, TX 77030, USA; Department of Integrative Biology and Pharmacology, The University of Texas Health Science Center at Houston, Houston, TX 77030, USA; Department of Integrative Biology and Pharmacology, The University of Texas Health Science Center at Houston, Houston, TX 77030, USA; Department of Integrative Biology and Pharmacology, The University of Texas Health Science Center at Houston, Houston, TX 77030, USA; Department of Integrative Biology and Pharmacology, The University of Texas Health Science Center at Houston, Houston, TX 77030, USA; Department of Integrative Biology and Pharmacology, The University of Texas Health Science Center at Houston, Houston, TX 77030, USA

**Keywords:** metabolic acidosis, NHE-3, AE1, proximal tubule, collecting duct, intercalated cells

## Abstract

Kidneys are central in maintaining acid-base homeostasis by recovering filtered bicarbonate (HCO_3_^−^) in the proximal tubule and by secreting H^+^ in the collecting duct. Here, we demonstrate a critical role of the exchange protein directly activated by cAMP (Epac) signaling, and particularly the Epac2, in governing renal adaptation to dietary acid load. RNAseq analysis of the renal cortical area revealed that Epac1&2 deficiency was associated with changes in gene profile seen in acidosis. Renal expression of Epac2 but not Epac1 was enhanced by acid load. Epac2-/- mice developed a pronounced metabolic acidosis due to the inability to acidify urine in response to dietary acid load. Deletion of Epac2 and Epac1 exerted additive inhibitory actions on expression of the Na^+^/H^+^ exchanger (NHE-3, *Slc9a3*) in the proximal tubule. Using super-resolution STED microscopy, we detected NHE-3 redistribution to the base of the brush border, which led to the impaired recovery after acidification in freshly isolated split-opened proximal tubules from Epac1&2-/- mice. Deletion of Epac2 but not Epac1 diminished H^+^ secretion in freshly isolated split-opened collecting ducts, compromised apical translocation of V-ATPase, and reduced anion exchanger 1 (AE1, *Slc4a1*) expression in the A-type intercalated cells, and caused lower levels of titratable acids in urine, whereas ammoniagenesis was not compromised. Overall, we demonstrate a previously unrecognized role of Epac signaling in renal adaptation to dietary acidification. While both Epac1 and Epac2 isoforms control NHE-3-dependent H^+^ secretion in the proximal tubule, only Epac2 is essential to augment H^+^ transport in the collecting duct to acidify urine.

## Graphical Abstract

**Graphical Abstract:**
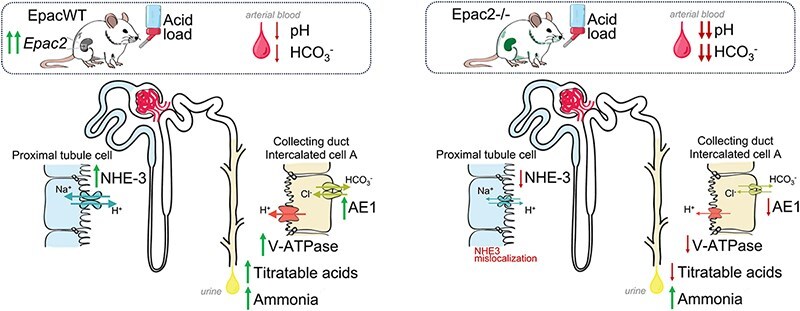

## Introduction

The maintenance of blood pH within a very narrow range around 7.4 has one of the highest priorities for proper whole-body health.^1^ Even small deviations from the physiological pH commonly seen, for instance, in metabolic acidosis, must be treated in clinical practice due to detrimental actions on bone and muscle metabolism and increased mortality rate.^2^ A typical Western diet, consisting of grains, meat, and dairy products, generates on average 70 mEq of acids on a daily basis.^2^ The kidneys play a chief role in maintaining acid-base balance by recovering the filtered bicarbonate and by excreting dietary acid load in the form of ammonium and titratable acids in urine.^1,3^ This is achieved by the regulation of transport rates of acids and bases by the proximal tubule and acid-secreting (A-type) intercalated cells of the distal nephron/collecting duct.^4,5^ Genetic or acquired deficiencies in the molecular transporting systems in either segment result in notable deviations of systemic acid-base homeostasis, as is manifested in the primary renal tubule acidosis (RTA) of distal (type 1), proximal (type 2) origin, as well as during generalized dysfunction of the proximal tubule, for instance, Fanconi syndrome.^3^ Moreover, the presence of metabolic acidosis directly correlates with the worsening of renal function in patients with chronic kidney disease and serves as a notable risk factor for mortality.^1,2^ While the end-effector membrane acid-base transporting systems, including but not limited to Na^+^/H^+^ exchanger (NHE-3, Slc9a3), Na^+^/HCO_3_^−^ cotransporter (NBCe1, Slc4a4), V-type H^+^-ATPase, anion exchanger 1 (AE1, Slc4a1), Cl^−^/HCO_3_^−^ exchanger (pendrin, Slc26a4), are generally well-characterized^3,6,7^; considerably less is known about signaling mechanisms governing the regulation of acid-base handling in both proximal and distal segments of the renal nephron during adaptation to a dietary acidic load.

Exchange protein directly activated by cAMP (Epac) is a largely underappreciated component of the complex cAMP signaling network.^8,9^ Epac signaling orchestrates a broad variety of cellular processes, including cell adhesion, MAPK/ERK activation, protein synthesis, mobilization of intracellular [Ca^2+^]_i_, endocytosis and exocytosis, to name a few.^10,11^ The cAMP-binding affinity of two known Epac isoforms, Epac1 and Epac2, is similar to that of protein kinase A (PKA), the “classical” cAMP target.^10^ However, it becomes increasingly appreciated that cAMP-triggered Epac- and PKA-dependent events do not commonly overlap.^12,13^ Cumulative experimental evidence supports the overall paradigm that Epac is an adaptive stress-response signaling cascade.^14^ In fact, deletion of individual or both Epac isoforms does not produce an overt adverse phenotype at baseline in mice. In contrast, over-activated Epac signaling has been reported in a variety of pathological states, including Alzheimer’s disease, cancer development and metastasis, atherosclerosis, heart failure, hyperalgesia, polycystic kidney disease, ischemic kidney injury, etc., reflecting most likely either compensatory or disease-driving actions.^15,16^

Epac1 and Epac2 have virtually identical biochemical properties in vitro with respect to cAMP binding and activation of the downstream effectors.^12,14^ However, the physiological functions of Epac1 and Epac2 are mostly non-redundant, largely because of their distinct tissue distributions.^14^ Furthermore, the non-overlapping subcellular localization of the two isoforms in the same tissue indicates that they are involved in different signalosomes, which orchestrate separate downstream processes.^17-19^ The kidney is a site with one of the highest Epac expression levels.^20^ Here, Epac1 and Epac2 expressions were detected in several tubular segments, most notably in the proximal tubule and collecting duct, with the expression pattern being similar between rodents and humans.^21^ We previously showed that deletion of either Epac isoform compromises renal Na^+^ transport in these segments by decreasing NHE-3 expression in the proximal tubule^22^ and by interfering with the upregulation of the epithelial Na^+^ channel (ENaC) activity following dietary Na^+^ restriction in the collecting duct.^23^ This, in turn, raises a question about the significance of Epac signaling and the supremacy of Epac isoforms in maintaining physiological pH and governing urinary acidification in response to dietary intake.

The current study was designed to investigate whether Epac signaling is instrumental in maintaining acid-base homeostasis and to assess pathophysiological ramifications upon deletion of a single or both Epac isoforms on systemic pH homeostasis and acid-base handling by the renal nephron in response to dietary acidification.

## Materials and Methods

### Reagents

All chemicals and materials were from Sigma (St. Louis, MO, USA), VWR (Radnor, PA, USA), Fisher (Waltham, MA, USA), and Tocris (Ellisville, MO, USA) unless noted otherwise and were at least of reagent grade.

### Research Animals

Animal use and welfare adhered to the NIH Guide for the Care and Use of Laboratory Animals following protocols reviewed and approved by the Animal Care and Use Committees of the University of Texas Health Science Center at Houston. For experiments, Epac WT (C57Bl/6), Epac1 -/-, Epac2 -/-, and Epac1&2-/- mice (8-12 wk) were used, as we described previously.^23^ In order to minimize sex-related variations in the measured experimental parameters, only males were used for experiments. Animals were housed in specialized rodent cages in a facility with a controlled 12-h light/dark cycle, temperature (23°C), air pressure, and humidity. Mice were fed standard rodent chow (Purina 5003) and had free access to tap water. For dietary acidification, animals were given water supplemented with 280 m m NH_4_Cl + 0.5% sucrose for 3 d, and the control group received vehicle (0.5% sucrose), as we did previously.^24^ To avoid bias, experiments were performed in a blind-folded manner (ie, the experimenter was not aware of the genotype of the tested mice).

### Systemic Measurements

Arterial blood samples (approximately 500 μL) were taken via terminal cardiac puncture in isoflurane anesthetized animals into heparinized syringes (Smiths Medical, Keene, NH, USA). The samples were immediately processed with an OPTI CCA-TS 2 blood gas and electrolyte analyzer (OPTI Medical Systems Inc., Roswell, GA, USA) supplied with E-Lyte CCA cassettes. Anion gap was calculated as the difference in measured cations (Na^+^ and K^+^) and anions (HCO_3_^−^ and Cl^−^) for each sample. Urinary pH was measured from 24-h urine collections under mineral oil in metabolic cages using an MI-410 pH microelectrode (Microelectrodes Inc., New Hampshire, NH, USA).

Urine titratable acid was measured using previously reported standard methods.^25^ In short, 24-h urine samples were acidified with 100 m m HCl, boiled for 2 min, and then cooled to 37°C. The volume of 100 m m NaOH required to titrate the sample back to pH 7.4 was measured. Distilled water samples were analyzed in parallel to calculate the background. Urine ammonium levels were measured in 24-h urine samples by an Ammonia Assay Kit (Abcam, United Kingdom; Cat.# ab102509) according to the manufacturer’s protocols.

### Mouse Kidney RNA Extraction and RNA-Seq Analysis

Total RNA was extracted using TRIzol™ Reagent (Invitrogen, Catalog Number 15596018) from equal amounts of mouse kidney cortex tissue containing ∼50 tubule segments predominantly proximal tubule and cortical collecting ducts mechanically isolated with watchmaker forceps under a stereomicroscope from EpacWT and Epac1&2-/- mice (3 animals per group) kept on a sodium deficient (<0.01% Na^+^; TD.90228 Envigo, Madison, WI, USA) diet for 1 wk. Total RNA was then purified and concentrated using the RNA Clean & Concentrator-5 Kit (Zymo Research, Catalog Number R1015), according to the manufacturer’s instructions. RNA concentration and purity were assessed using a ThermoFisher NanoDrop spectrophotometer. RNA-seq and differential expression analysis were performed by Novogene. Enrichment analyses were performed using the Toppgene Suite.^26^

### Isolation and Split-Opening of Renal Tubule Segments

The procedure for isolating the cortical collecting ducts from mouse kidneys suitable for fluorescent pH_i_ measurements, closely followed the protocols previously published by our group.^24,27^ Kidneys were cut into thin slices (<1 mm), which were then placed into an ice-cold solution containing (in m m): 150 NaCl, 5 KCl, 1 CaCl_2_, 2 MgCl_2_, 5 glucose, and 10 HEPES (pH 7.35). The cortical collecting ducts were mechanically isolated by micro-dissection from straight cortical-to-medullary sectors, containing approximately 30-50 renal tubules, using watchmaker forceps under a stereomicroscope. Cortical collecting ducts were visually identified by their morphological features (pale color; coarse surface) and by their post-experimental staining with antibodies against the AQP2 water channel, a marker of collecting duct principal cells.

For isolation of proximal tubules, kidneys were similarly cut into thin slices (<1 mm) with slices placed into an ice-cold solution containing (in m m): 130 NaCl, 20 NaHCO_3_, 5 KCl, 1 CaCl_2_, 2 MgCl_2_, 5 glucose, and 10 HEPES (pH 7.35). Small cortical regions were mechanically dissected and transferred to the solution containing 0.8 mg/mL collagenase type I (Alfa Aesar, Ward Hill, MA 01835) for 9 min at 37°C, followed by an extensive washout with control solution at room temperature. Proximal tubules were identified as the segments immediately following the initial S-shape convolution after the glomerular capsule, thereby predominantly representing the S2-S3 segments. The PT origin was further verified by positive staining with antibodies against the AQP1 water channel (now shown).

Individual proximal tubules or cortical collecting ducts were attached to 5×5 mm cover glasses coated with poly-L-lysine. The cover-glass was then placed into a recording chamber mounted on an inverted Nikon Eclipse Ti-S microscope and permanently perfused at room temperature. Proximal tubules or cortical collecting ducts were further split-opened with 2 sharpened micropipettes, controlled with different micromanipulators, to reliably monitor pH_i_ signals from individual cells within a monolayer. The segments were used within 2 h of their isolation.

### Intracellular pH Measurements

Split-opened proximal tubules or cortical collecting ducts were loaded with acetoxymethyl ester (AM) or 2“,7”-Bis-(2-Carboxyethyl)-5-(and-6)-carboxyfluorescein (BCECF) by incubation with 15 μm BCECF-AM in the bath solution for 40 min at room temperature, followed by a washout with the bath solution for an additional 10 min. The segments were placed in an open-top imaging study chamber (RC-26GLP; Warner Instruments, Hamden, CT, USA) with a bottom coverslip viewing window and the chamber attached to the microscope stage of a Nikon Ti-S Wide-Field Fluorescence Imaging System (Nikon Instruments, Melville, NY, USA) integrated with a Lambda XL light source (Sutter Instrument, Novato, CA, USA) and QIClick 1.4 megapixel monochrome CCD camera (QImaging, Surrey, bc, Canada) controlled via NIS Elements 4.3 Imaging Software (Nikon Instruments, Melville, NY, USA). Cells were imaged with a 40X Nikon Super Fluor objective, and regions of interest (ROIs) were drawn around individual cells. The BCECF fluorescence intensity ratio was determined by excitation at 495 nm and 440 nm, and by calculating the ratio of the emission intensities at 520 nm every 5 s. The changes in the ratio were converted into changes in pH_i_ by performing a calibration in high K^+^ solutions (145 m m KCl) with predefined pH (6.0, 7.0, and 8.0, adjusted by HCl and KOH, respectively) in the presence of 15 µm nigericin, as was originally described.^28^ The calibration was calculated for each tubular segment individually. Minor BCECF bleaching during the timeline of experiments was corrected, as necessary.

Experiments were performed under continuous perfusion of a solution containing (in m m): 130 NaCl, 20 NaHCO_3_, 5 KCl, 1 CaCl_2_, 2 MgCl_2_, 5 glucose, and 10 HEPES for the proximal tubules (HCO_3_^−^ is present) and 150 NaCl, 5 KCl, 1 CaCl_2_, 2 MgCl_2_, 5 glucose, and 10 HEPES (HCO_3_^−^ is absent) for the collecting ducts at 1.5 mL/min rate. The rationale is that HCO_3_^−^ is reabsorbed in the proximal tubule and the thick ascending limb with little or no HCO_3_^−^ reaching the collecting duct. In our test experiments, equimolar addition of HCO_3_^−^ did not affect the experimental outcomes in cortical collecting ducts obtained in the absence of HCO_3_^−^. For the intracellular acidification protocol, a solution containing 40 m m NH_4_Cl (equimolar substitution of NaCl in respective bath solutions for proximal tubule and collecting duct) was applied to the recording chamber for 2 min. The rate of recovery after acidification was calculated as a linear slope of the initial pH_i_ recovery rate (typically 30 s) from the lowest pH_i_ values for each cell after removal of the acidification pulse, as we did previously.^24,25,27^ Acid-secreting (A-type) and base-secreting B-type collecting duct intercalated cells were distinguished by their opposite changes in pH_i_ levels (alkalization and acidification) in response to application of the basolateral ClC-K2 Cl^−^ channel blocker, 5-Nitro-2-(3-phenylpropylamino) benzoic acid (NPPB, 100 µm), as we have previously described.^24^ On average, 5 individual segments from 3 different mice were used for each experimental set. There were approximately equal numbers of cells per tested segment with no apparent heterogeneity in cellular responses between the tested segments within a group.

### Western Blotting

Immediately after dissection, kidneys were placed on ice, decapsulated, and homogenized in one of two ice-cold lysis buffers. For Epac1 and Epac2 antibodies, a hypotonic buffer (TrisCl, EDTA, and 1% Triton X-100) was used. For Pendrin, NHE-3, AE1, and NBCe1 antibodies, a 5% Sorbitol buffer (sorbitol, histidine-imidazole, and Na_2_EDTA) was used. Both buffers were supplemented with Complete Mini protease and PhosSTOP phosphatase inhibitor cocktails (Roche Diagnostics, USA). The homogenates were centrifuged at 2000 *g* for 15 min at +4°C, and the sediment was discarded. Protein concentration was determined using a NanoPhotometer N60. The samples (50 μg/lane) were separated on 9% polyacrylamide gels at 150 V for 75 min and transferred to a nitrocellulose membrane for 110 min at 100 V. Equal protein loading was verified by Ponceau red staining, performed using standard procedures. Nitrocellulose membranes were incubated with primary antibodies overnight at 4°C: anti-Epac1 (mouse mAb, 1:1000 Cell Signaling Technology, USA; Cat. #4155), anti-Epac2 (rabbit mAb, 1:250 Cell Signaling Technology, USA; Cat. #43239), anti-NHE-3 (rabbit mAb, 1:1000 Alomone Labs, Israel; Cat. #ANX-033), anti-NBCe1 (rabbit mAb, 1:1000 Alomone Labs, Israel; Cat. #ANT-07), anti-AE1 (rabbit mAb, 1:1000 Invitrogen, USA; Cat. # PA5-80030), or anti-pendrin (rabbit mAb, 1:1000 Invitrogen, USA; Cat. # PA5-42060). Following washout (3 times for 10 min in TBS-Tween), the membrane was incubated with peroxidase-conjugated goat anti-rabbit or anti-mouse (1:10 000, Jackson ImmunoResearch Laboratories, USA) secondary antibodies for 1 h at room temperature. Blots were quantified using ImageJ 1.50 software (NIH, USA). The intensity of each protein band was normalized to the total signal of its respective lane, as measured by Ponceau red staining.

### Immunofluorescent Microscopy (Kidney Sections)

Freshly isolated kidneys were decapsulated, fixed in 10% neutral buffered formalin at +4°C overnight, and subsequently soaked in 0.9 m sucrose overnight at +4°C. Next, the kidneys were placed into cryo-tubes with embedding medium (Andwin Scientific Tissue-Tek™ CRYO-OCT Compound 4583, Torrance, CA, USA; Cat. #14-373-65) and flash-frozen in liquid nitrogen for 3-5 min. Transverse cut 6 µm-thick sections were made on a CM 1850 cryostat (Leica, Buffalo Grove, IL, USA). The sections were allowed to warm to room temperature, washed with PBS and incubated with low-pH retrieval solution (Thermo Scientific, Pittsburg, PA, USA; Cat. #00-4955-58) for 30 min at 60°C. After washing with mQ water, the sections were permeabilized with 0.1% Triton X-100 (Sigma-Aldrich, St. Louis, MO, USA; Cat. #56H0850) for 15 min. After an extensive washout, the samples were treated with 10% normal goat serum for 1 h at room temperature. Sections were incubated overnight at +4 °C with primary antibodies: rabbit anti-mouse anti-NHE-3 (1:25, Abcam, United Kingdom; Cat. #ab95299), rabbit anti-mouse anti-AE1 (1:200, Invitrogen, USA; Cat. #PA5-80030), or rabbit anti-mouse anti-a4 V-ATPase (1:200 Proteintech, Rosemont, IL, USA Cat. #21570-1-AP) antibodies. Following a 20-min washout with PBS at room temperature, the samples were incubated with goat anti-rabbit Alexa 647 (H + L) secondary antibodies (1:1500, Thermo Scientific, Pittsburg, PA, USA; Cat. #A32733) or goat anti-rabbit StarRed (1:500, Abberior Instruments America LLC, USA; Cat. #STRED-1002) for 75 min at room temperature. Following a 20-min washout with PBS at room temperature, the samples were incubated with 5% normal rabbit serum for 30 min at room temperature and, subsequently, with rabbit anti-mouse anti-AQP2-ATTO Fluor-550 (1:200, Alomone Labs, Israel; Cat. #AQP2-002-AO), or mouse monoclonal anti-villin conjugated with Alexa Fluor 546 (1:50, Santa Cruz Biotechnology Inc., Dallas, TX, USA; Cat. #sc-58897) antibodies overnight at +4°C. Nuclei were stained with DAPI (0.5 µg/mL) for 15 min at room temperature. The samples were mounted with ProLong Gold antifade reagent (Thermo Scientific, Pittsburgh, PA, USA; Cat. #P10144). *Confocal microscopy*: Fluorescence microscopy was performed at the Center for Advanced Microscopy, a Nikon Center of Excellence, at McGovern Medical School, UTHealth Houston. The labeled kidney sections were imaged with a Nikon A1R confocal microscope, as similarly described previously.^29^ Briefly, samples were excited with 405, 640, and/or 561 nm laser diodes, and emission was captured with a 16-bit Cool SNAP HQ2 camera (Photometrics) interfaced to a PC running NIS Elements software. *STED super-resolution microscopy* was performed at the Center for Advanced Microscopy, a Nikon Center of Excellence, at McGovern Medical School, UTHealth Houston. Images were acquired with an Abberior Facility Line 3D-STED microscope equipped with a 775 nm (2750 mW, 40 MHz) depletion laser. The samples were excited with 405 (Dapi) and 640 (StarRed-NHE-3) nm laser diodes. The signal was collected using Dual MATRIX Spectral Detectors and the 60x oil objective. Initially, the confocal images were acquired where the ROI around transverse-oriented proximal tubules were selected for STED-based enhancement. Images were acquired with a resolution of 10 nm per pixel of approximately 35 × 35 µm regions. For analysis of NHE-3 distribution, average intensity was quantified within two 1×1 µm regions, one at the tip and one at the base of the brush border, in individual proximal tubule cells.

### Immunofluorescent Microscopy (Split-Opened Collecting Ducts)

Following pH_i_ measurements, split-opened collecting ducts were fixed with 10% neutral buffered formalin (AzerScientific, Morgantown, PA, USA; Cat. #PFNBF240) for 15 min at room temperature. After fixation, the samples were permeabilized by the addition of 1% SDS (Sigma-Aldrich, St. Louis, MO, USA; Cat. #L4390) in phosphate buffer solution (PBS) for 10 min and washed in PBS 2 times for 10 min. Nonspecific staining was blocked with 10% normal goat serum (Novus Biologicals™, Centennial, CO, USA; Cat. #NBP223475) in PBS for 1 h at room temperature. The samples were incubated overnight at + 4°C in the dark with rabbit anti-mouse anti-AQP2 antibodies (1:4000, Alomone Labs, Israel; Cat. #AQP2-002), washed with PBS, and incubated with goat anti-rabbit Alexa 594 F(ab’) secondary antibodies (1:1000 Jackson Immunoresearch, West Grove, PA, USA Cat. #111-587-003) for 60 min at room temperature. After washing with PBS (2 times for 5 min), the samples were stained with 4',6-diamidino-2-phenylindole (DAPI) (300 n m concentration, MilliporeSigma™, Calbiochem, San Diego, CA, USA; Cat. #5.08741.0001) to visualize nuclei. The samples were mounted with Fluoromount mounting media (Thermo Scientific, Pittsburgh, PA, USA). The samples were imaged with a Nikon A1R confocal microscope using 405 and 561 nm laser diodes and emission captured with a 16-bit Cool SNAP HQ2 camera (Photometrics) interfaced to a PC running NIS Elements software. Maximal intensity projections were created from serial confocal images with a 0.75 µm increment along the vertical axis.

### Data Analysis

All data are reported as mean ± SEM or ± SD, as indicated, with individual measurements shown for each tested group. Statistical comparisons were made using one-way (single variable) or two-way (multiple variables: genetic background, acid load) ANOVA with a post-hoc Tukey test or a one-way repeated measures ANOVA with a post hoc Bonferroni test, as appropriate. A *P*-value less than 0.05 was considered significant. All data analysis was performed using OriginPro 2016. For normality, the Shapiro-Wilk test was utilized. Data were considered normal when the *P*-value was greater than 0.05; otherwise, a Wilcoxon Rank-Sum nonparametric test was used to assess a significant difference.

## Results

### Deletion of Epac Signaling Exacerbates Diet-Induced Metabolic Acidosis

**Figure 1.**
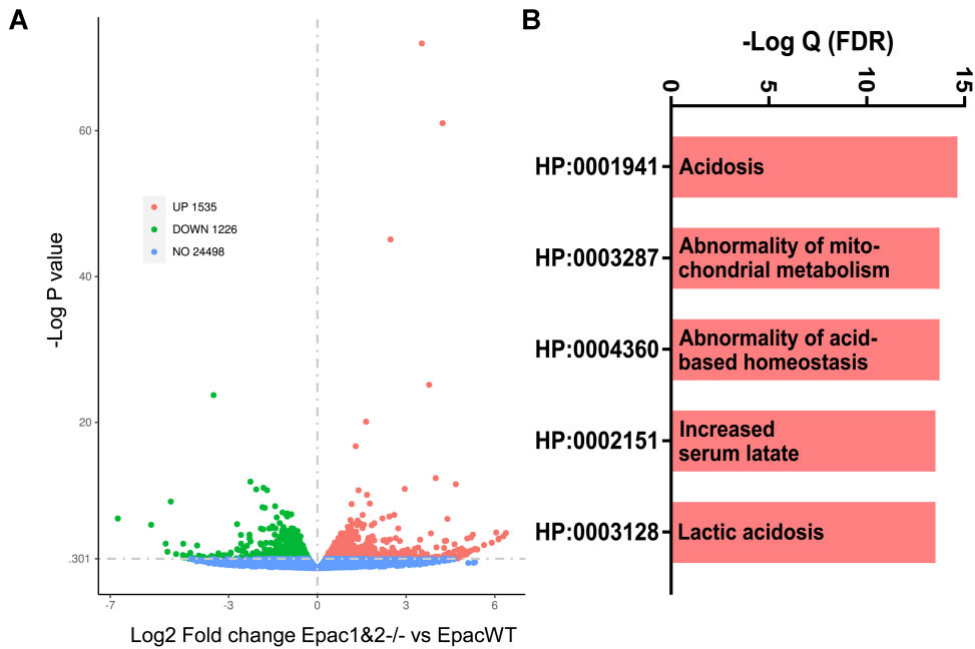
Upregulation of gene transcripts is associated with acidosis in Epac1&2-/- mice. (A) Volcano plot of RNA-seq data from cortical/outer-medullary kidney samples isolated from Epac1&2-/- and EpacWT mice fed a Na^+^ deficient diet for 1 wk. (B) The top five enriched human genotype Gene Ontology (GO) of upregulated genes in Epac1&2-/- versus WT from the data in panel A.

We previously documented that Epac1 and Epac2 regulate Na^+^ reabsorption in the proximal tubule and collecting duct, with their deletion causing natriuresis due to the impaired adaptation to dietary Na^+^ deficiency.^22,23^ To gain insights into the overall changes in the gene expression profile, we first performed RNAseq analysis in cortical regions of kidneys containing ∼50 tubule segments, predominantly proximal tubules and collecting ducts isolated from WT and Epac1&2-/- mice maintained on a Na^+^-deficient diet. As shown in the volcano plot in Figure 1A, deletion of both Epac isoforms led to a significant upregulation of 1535 transcripts and downregulation of 1226 transcripts. Gene Ontology (GO) enrichment analysis of the 1535 upregulated genes revealed that the top five most enriched GO sets associated with the human phenotype are all related to acidosis (Figure 1B). This raises the possibility that Epac signaling contributes to pH homeostasis by regulating acid-base transport in the renal nephron.

**Figure 2.**
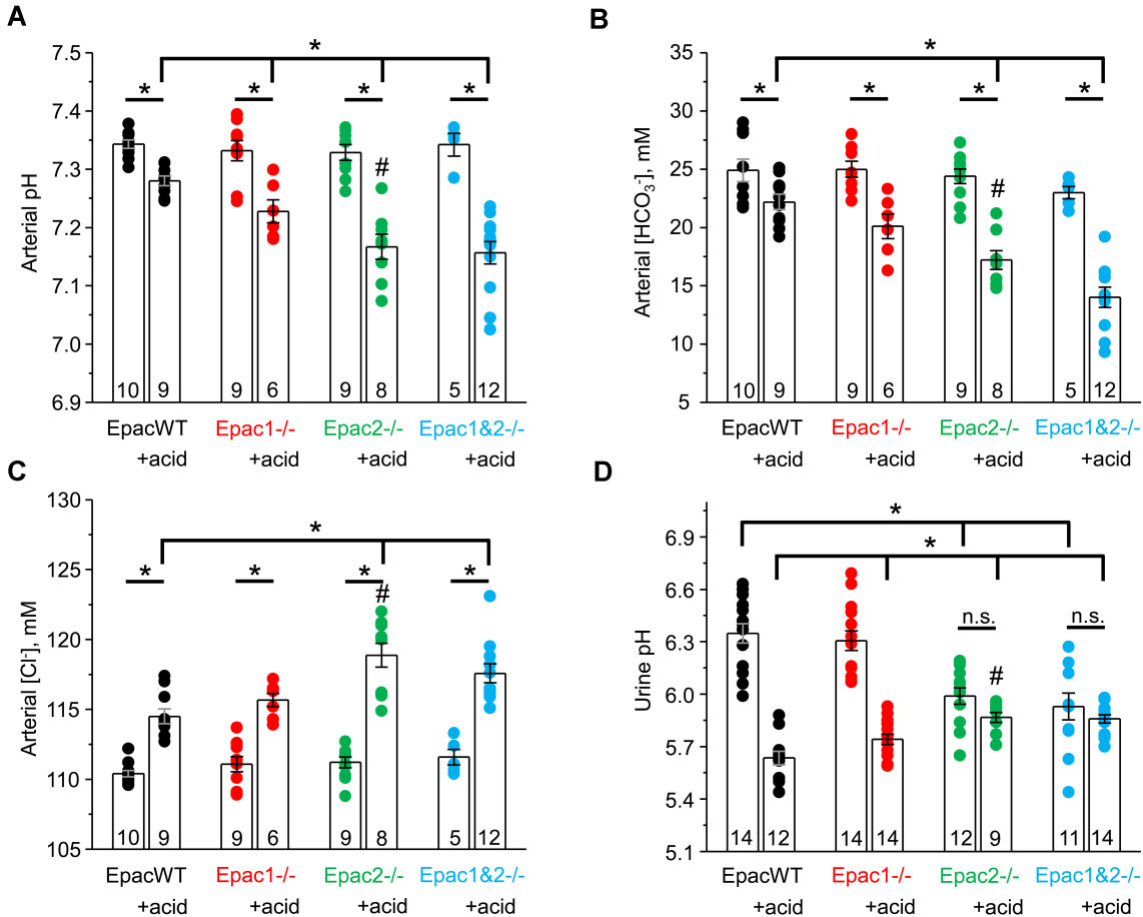
Deletion of Epac1 (less) and Epac2 (more) exacerbates dietary-induced metabolic acidosis. Summary graphs of arterial pH (A), HCO_3_^−^ (B), Cl^−^ (C), and 24-h urinary pH (D) levels in EpacWT (black), Epac1-/- (red), Epac2-/- (green), and Epac1&2-/- (blue) mice given 0.5% sucrose (vehicle) and 280 m m NH_4_Cl + 0.5% sucrose in drinking water for 3 d (+acid). Individual values are shown with circles. Numbers of individual mice are shown for each group. n.s.—not significant; *—significant difference (*P* < 0.05, one-way ANOVA with post-hoc Tukey test) between groups as indicated with lines and brackets on the top. #—significant difference (*P* < 0.05, one-way ANOVA with post-hoc Tukey test) versus Epac1-/- + acid group. Two-way ANOVA with post-hoc Tukey test shows significant (*P* < 0.05) differences when considering genotype and dietary intervention and their significant interaction for panels A-D.

**Figure 3.**
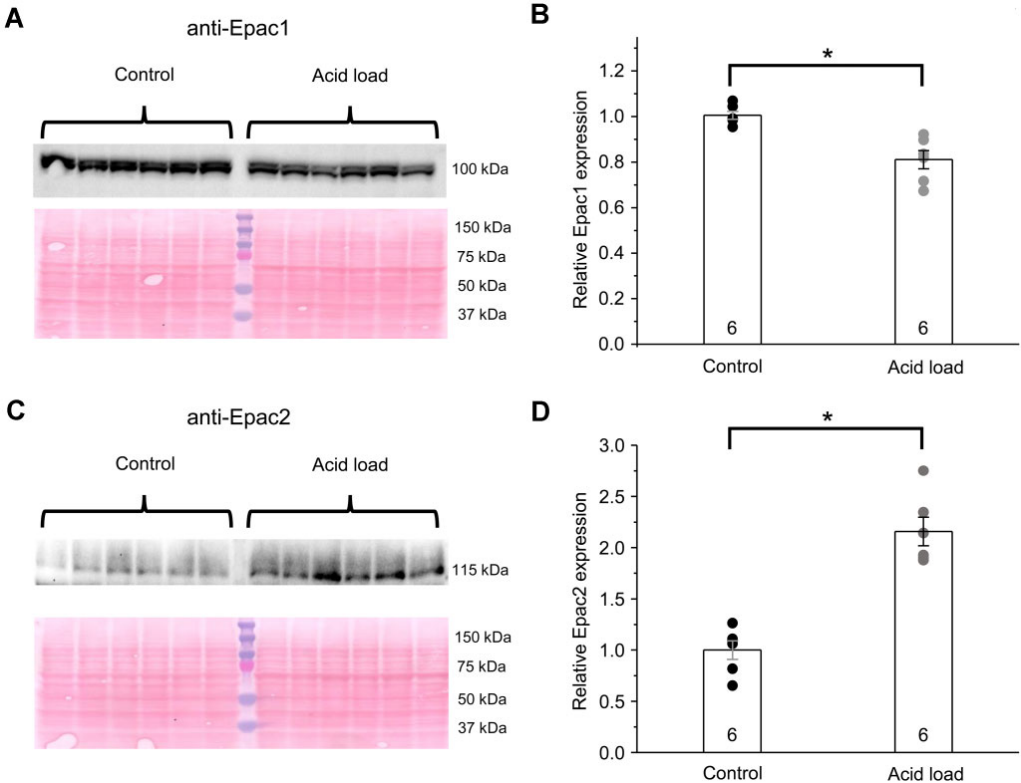
Renal Epac2 expression is augmented during metabolic acidosis. (A) Representative Western blot probed with anti-Epac1 antibodies from whole kidney lysates of EpacWT mice given 0.5% sucrose vehicle (control) and 280 m m NH_4_Cl + 0.5% sucrose in drinking water for 3 d (acid load), as indicated with brackets on the top. Each line represents an individual animal. The Ponceau red staining of the same nitrocellulose membrane is shown below to demonstrate equal protein loading. (B) Summary graph comparing Epac1 expression from the Western blot shown in panel A. The intensity values were normalized to the total signal of the respective lanes in Ponceau red staining. *—significant decrease (*P* < 0.05; one-way ANOVA with post-hoc Tukey test) versus control. (C) Representative Western blot probed with anti-Epac2 antibodies from whole kidney lysates of EpacWT mice given 0.5% sucrose vehicle (control) and 280 m m NH_4_Cl + 0.5% sucrose in drinking water for 3 d (acid load), as indicated with brackets on the top. Each lane represents an individual animal. Ponceau red staining of the same nitrocellulose membrane is shown below to demonstrate equal protein loading. (D) Summary graph comparing Epac2 expression from the Western blot shown in panel A. The intensity values were normalized to the total signal of the respective lanes in Ponceau red staining. *—significant decrease (*P* < 0.05; one-way ANOVA with post-hoc Tukey test) versus control.

Thus, we next assessed the pathophysiological consequences of deleting a single or both Epac isoforms on acid-base balance at the whole-body level. Epac1-/- and Epac2-/- mice are moderately polyuric at baseline,^22^ which complicates administering a comparable acid load via drinking water. Interestingly, we found that switching to acid-containing water abolished differences between Epac-deficient strains and EpacWT mice during the treatment period (Supplementary Figure S1). In fact, similar 24-h water intake (Panel A) and urine output (Panel B) between Epac WT, Epac1-/-, Epac2-/-, and Epac1&2-/- mice during treatment days 1-3 provide strong support for a comparable acid load in all tested strains. There were no significant differences in arterial pH (Figure 2A), arterial HCO_3_^−^ (Figure 2B), and arterial Cl^−^ (Figure 2C) levels between EpacWT, Epac1-/-, Epac2-/-, and Epac1&2-/- mice at the baseline. Interestingly, we detected significantly lower urinary pH values in Epac2-/- and Epac1&2-/- mice, whereas no differences were found between Epac1-/- and EpacWT (Figure 2D). Dietary acidification with NH_4_Cl supplementation in drinking water for 3 d led to only a mild though significant reduction in pH and HCO_3_^−^ with a respective rise in Cl^−^ levels in EpacWT mice (Figure 2A-C), thus producing hyperchloremic metabolic acidosis, as expected. Importantly, deletion of Epac1, Epac2, and Epac1&2 significantly decreased arterial pH compared to values seen in Epac WT after dietary acidification: 7.23 ± 0.02 (*P* < 0.05), 7.17 ± 0.02 (*P* < 0.05), and 7.16 ± 0.02 (*P* < 0.05) versus 7.28 ± 0.02, respectively (Figure 2A). Arterial HCO_3_^−^ levels were 20.1 ± 1.1 m m, 17.2 ± 0.8 m m (*P* < 0.05), and 14.0 ± 0.9 m m (*P* < 0.05) versus 22.2 ± 0.7 m m in Epac WT, respectively (Figure 2B). This was associated with corresponding significant increases in Cl^−^ levels in all groups (Figure 2C). Bivariate analysis (two-way ANOVA) revealed a significant interaction between genotype and treatment in the tested parameters, suggesting the importance of intact Epac signaling in adaptation to dietary acidification. Moreover, the effect of Epac2 deletion was significantly stronger than the effect of Epac1 deletion (Figure 2A-C). There were no significant changes in plasma K^+^ values for all tested groups, and only a small increase in plasma Na^+^ in acid-loaded groups, as shown in Supplementary Figure S2. There were no changes in anion gap for Epac1-/- (17.5 ± 1.1 m m versus 14.3 ± 1.1 m m), Epac2-/- (18.6 ± 1.1 m m versus 16.3 ± 1.1 m m), and Epac1&2-/- (19.3 ± 1.3 m m versus 17.2 ± 0.5 m m) after acid load compared to baseline conditions, respectively. Moreover, these values were comparable with the values seen in Epac WT (17.0 ± 0.5 m m upon acid load versus 16.2 ± 0.8 m m in control). Of note, Epac2-/- and Epac1&2-/- mice failed to decrease urinary pH in response to dietary acidification, indicating impaired H^+^ secretion in the distal nephron. In contrast, Epac1-/- mice were able to reduce urinary pH, though, to a lower extent when compared to the values observed in EpacWT (Figure 2D). Importantly, dietary acidification led to a mild but significant, decrease in renal Epac1 expression (Figure 3A, B), whereas Epac2 levels were elevated 2-fold in acid-loaded mice (Figure 3C, D). This provides a mechanistic insight into the milder phenotype of Epac1-/- versus Epac2-/- mice with respect to acid-base balance. Taken together, the results in Figures 2 and 3 demonstrate a critical role for Epac signaling in adaptation to dietary acid load, with Epac2 isoform making the dominant contribution.

### Epac1 and Epac2 Regulate Functional NHE-3 Expression in the Proximal Tubule in an Additive Manner

**Figure 4.**
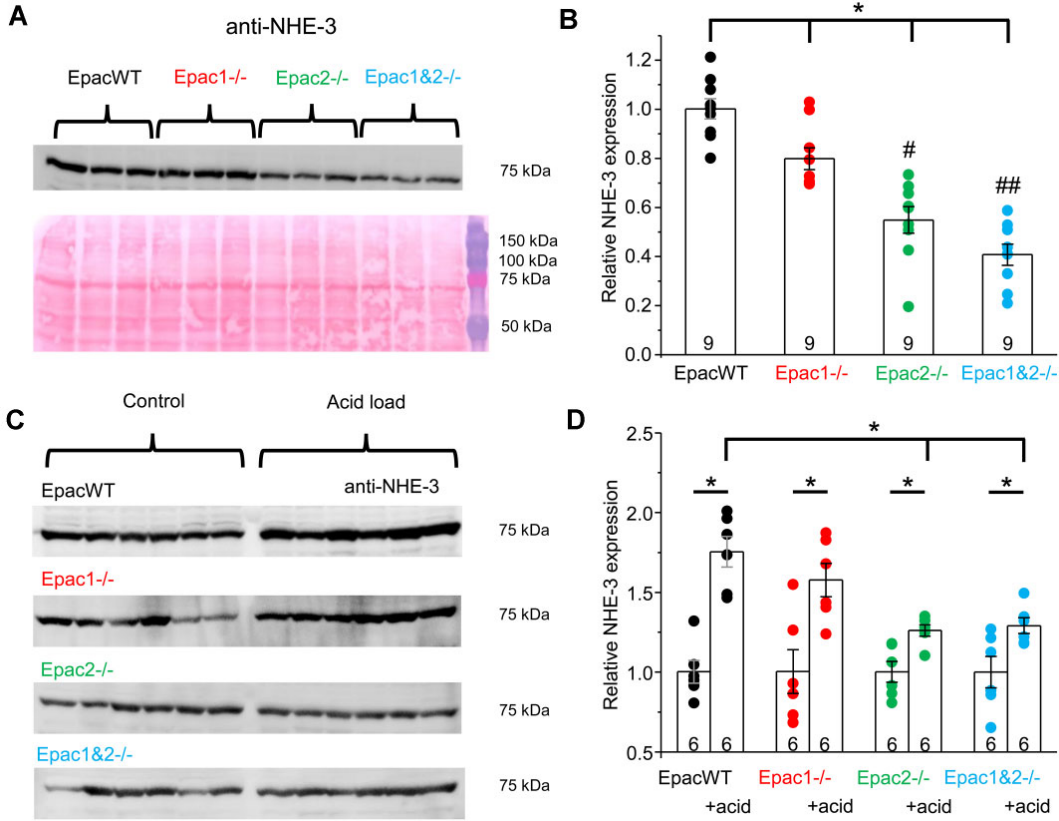
Deletion of Epac isoforms decreases NHE-3 expression at baseline and during metabolic acidosis. (A) Representative Western blot probed with anti-NHE-3 antibodies from whole kidney lysates of EpacWT, Epac1-/-, Epac2-/-, and Epac1&2-/- mice at baseline, as indicated with brackets on the top. Each lane represents an individual animal. Ponceau red staining of the same nitrocellulose membrane is shown below to demonstrate equal protein loading. (B) Summary graph comparing NHE-3 expression from the Western blots similar to those shown in panel A. The intensity values were normalized to the total signal of the respective lanes in Ponceau red staining. *—significant difference (*P* < 0.05, one-way ANOVA with post-hoc Tukey test) between groups as indicated with lines and brackets on the top. #—significant difference (*P* < 0.05, one-way ANOVA with post-hoc Tukey test) versus Epac1-/- group. ##—significant difference (*P* < 0.05, one-way ANOVA with post-hoc Tukey test) versus Epac1-/- and Epac2-/- groups. (C) Representative Western blots probed with anti-NHE-3 antibodies from whole kidney lysates of EpacWT, Epac1-/-, Epac2-/-, and Epac1&2-/- mice given 0.5% sucrose vehicle (control) and 280 m m NH_4_Cl + 0.5% sucrose in drinking water for 3 d (acid load), as indicated with brackets on the top. Each line represents an individual animal. The respective Ponceau red staining demonstrating equal protein loading is shown in the Supplement. (D) Summary graph comparing NHE-3 expression from the Western blots similar to those shown in panel C. The intensity values were normalized to the respective NHE-3 values in control for each genotype separately. *—significant difference (*P* < 0.05, one-way ANOVA with post-hoc Tukey test) between groups as indicated with lines and brackets on the top.

It is generally accepted that NHE-3-dependent luminal H^+^ secretion is the first critical step in the recovery of the filtered HCO_3_^−^ by the proximal tubule.^3,6,30^ Thus, we next examined the functional consequences of Epac isoform ablation on NHE-3 expression, localization, and activity. As shown in the representative Western blot from whole kidney lysates in Figure 4A and the corresponding summary graph in Figure 4B, deletion of either Epac1 or Epac2 significantly decreases NHE-3 levels at baseline. A statistically stronger effect (*P* = 0.002) was observed for Epac 2 deletion. Moreover, the concomitant deletion of Epac1 and Epac2 further significantly decreased NHE-3 levels, when compared to the values seen in single knockouts (*P* < 0.001 for Epac1-/- and *P* = 0.05 for Epac2-/-). We next tested whether Epac signaling is necessary for the upregulation of NHE-3 expression during metabolic acidosis. As shown in the Western blots (Figure 4C) and respective summary graph (Figure 4D), dietary acidification significantly increased NHE-3 levels in EpacWT and all Epac-deficient strains. However, the magnitude of NHE-3 potentiation was similar in EpacWT and Epac1-/- (75 ± 9% and 58 ± 10%, respectively), whereas it was markedly blunted in Epac2-/- and Epac1&2-/- (26 ± 4% and 29 ± 5%, respectively). Taken together, these results suggest that Epac1 and Epac2 regulate renal NHE-3 expression in an additive manner, with Epac2 having a stronger contribution. Moreover, Epac2, but not Epac1, facilitates upregulation of NHE-3 levels in response to dietary acidification.

**Figure 5.**
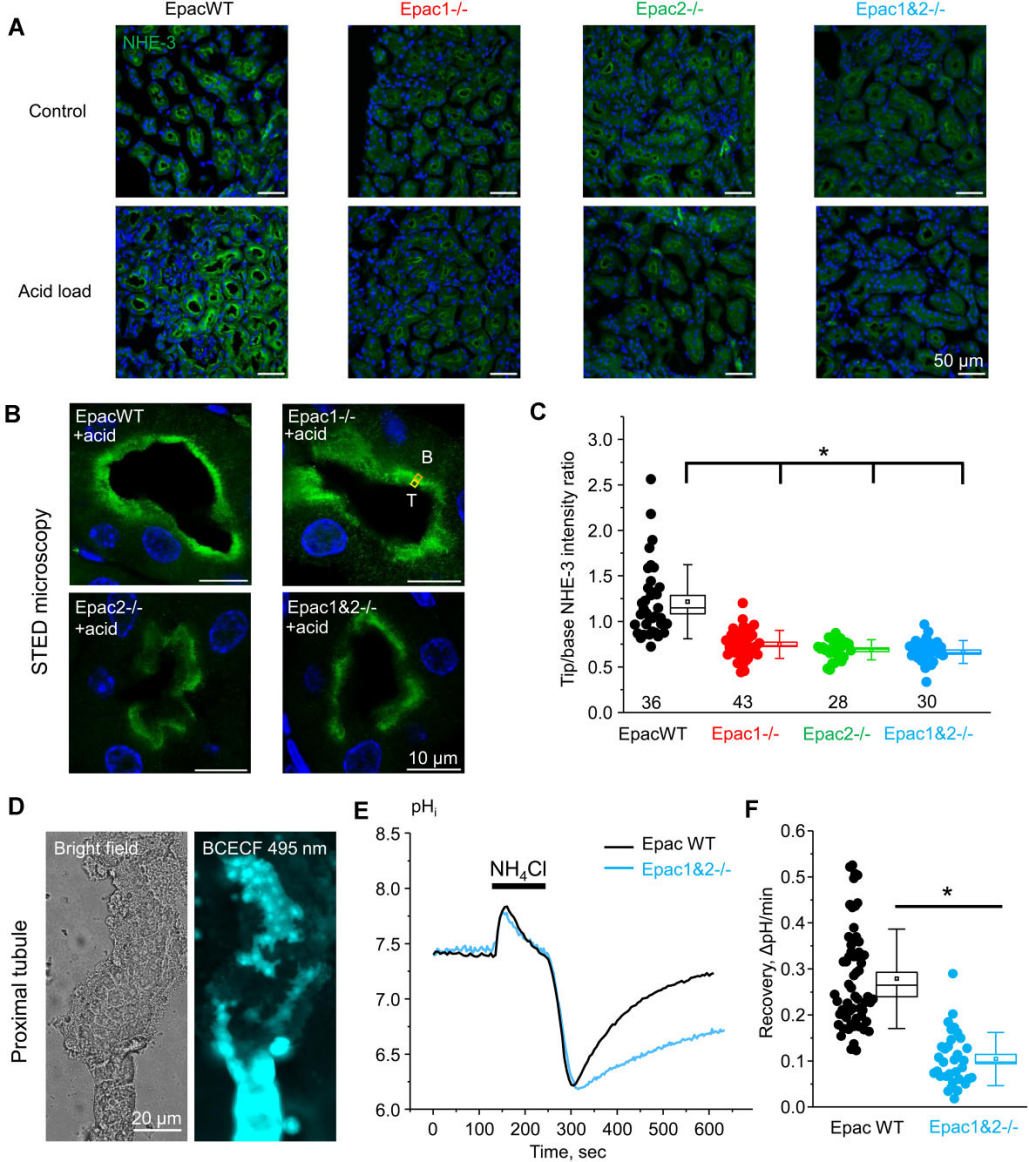
Deletion of Epac isoforms compromises subcellular NHE-3 translocation and activity in response to dietary acidification. (A) Representative confocal images of renal cortical area from kidney sections probed with anti-NHE-3 (pseudocolor green) in EpacWT, Epac1-/-, Epac2-/-, and Epac1&2-/- mice given 0.5% sucrose vehicle (control) and 280 m m NH_4_Cl + 0.5% sucrose in drinking water for 3 d (acid load). All images were taken with identical laser intensity settings for each wavelength. Nuclear DAPI staining is shown in pseudocolor blue. (B) Representative super-resolution STED images of individual proximal tubules probed with anti-NHE-3 (pseudocolor green) in EpacWT, Epac1-/-, Epac2-/-, and Epac1&2-/- mice given 280 m m NH_4_Cl + 0.5% sucrose in drinking water for 3 d. (C) Summary graph comparing the ratio of averaged fluorescent intensities in identical-sized boxes (1 × 1 µm) positioned on the tip (T) and base (B) areas of the brush border, as highlighted in panel B. Individual data points are shown as circles. At least 4 individual kidney sections were analyzed for each group. Means are shown with dots, medians are indicated by lines, bars represent standard error, and whiskers define standard deviation. *—significant difference (*P* < 0.05, Wilcoxon Rank-Sum nonparametric test) versus EpacWT, as indicated with a bracket on the top. (D) Representative images of a split-opened proximal tubule loaded with pH-sensitive dye (BCECF) are shown under bright-field illumination and upon fluorescent excitation of BCECF at 495 nm. (E) Representative time courses of changes in pH_i_ in individual proximal tubule within a split-opened area from WT (black) and Epac1&2 -/- (blue) mice in response to intracellular acidification after 40 m m NH_4_Cl (shown with a black bar on top). (F) Summary graph showing the recovery after acidification rates with NH_4_Cl in proximal tubule cells from the experiments in panel E. The values were calculated for each individual cell (shown as dots) as a linear slope of the initial pH_i_ recovery from the lowest pH_i_ value after 40 m m NH_4_Cl removal. Proximal tubules from 3 different mice were analyzed for each group. Means are shown with dots, medians are indicated by lines, bars represent standard error, and whiskers define standard deviation. *—significant decrease (*P* < 0.05, Wilcoxon Rank-Sum nonparametric test) versus EpacWT, as indicated with a line on the top.

We next used immunofluorescent confocal microscopy to test whether Epac-isoform deletion affects subcellular NHE-3 distribution. As shown on the representative images, the fluorescent signal reporting on NHE-3 was similarly localized to the brush border apical region in all tested groups at baseline, while the intensity of the signal was drastically reduced in Epac knockouts using the same laser settings (Figure 5A). Furthermore, dietary acid load caused a pronounced upregulation of the NHE-3-reporting fluorescent signal in the apical region in EpacWT mice, as anticipated. In contrast, we did not observe a similar upregulation of the NHE-3 signal in Epac-deficient mice subjected to dietary acid load (Figure 5A). Previously published evidence suggests that redistribution of NHE-3 from the tip to the base of the brush border of proximal tubule cells is associated with its inactivation.^31,32^ To better resolve the subcellular localization and probe for potential mislocalization of NHE-3, we next utilized super-resolution STED microscopy. As Supplementary Figure S3A-C shows, this provided better spatial resolution and a higher signal-to-noise ratio. Almost ideal co-localization was observed between the NHE-3-reporting signal and the brush border marker villin in EpacWT mice after acid load (Supplementary Figure S3D). In contrast, the NHE-3-reporting signal is shifted towards the base of the brush border in Epac-deficient mice, with a stronger effect seen in Epac2-/- and Epac1&2-/- mice on this condition. Thus, we next quantified the ratio of signal intensities in 1 × 1 μm areas located at the tip and at the base of the brush border (Figure 5B). As summarized in Figure 5C, the intensity of the fluorescent signal is higher in the luminal inner region (T, tip of brush border) in Epac WT, whereas the signal is shifted towards the outer region (B, base of brush border) similarly in Epac1-/-, Epac2-/-, and Epac1&2-/- mice.

We next explored whether reduced NHE-3 expression and its altered brush border localization are also associated with an impaired H^+^ secretion by the proximal tubule cells in Epac deficient mice. For this, we monitored changes in pH_i_ in freshly isolated split-opened proximal tubules from EpacWT and Epac1&2-/- mice loaded with a pH-sensitive dye, BCECF (Figure 5D). An acute intracellular acidification was induced with the standard ammonium pulse protocol.^33^ As shown in Figure 5E, the application of 40 m m NH_4_Cl for 2 min caused a transient alkalization. Removal of the NH_4_Cl was followed by an acidification and subsequent pH_i_ recovery, which reflects the rate of H^+^ secretion. As summarized in Figure 5F, the rate of recovery from NH_4_Cl-induced acidification was significantly (*P* < 0.001) lower in Epac1&2-/- mice (0.10 ± 0.01 ΔpH/min) compared to EpacWT (0.28 ± 0.01 ΔpH/min).

Taken together, our results in Figures 4-5 show a major role of Epac signaling in the regulation of NHE-3 expression, localization, and activity with a greater contribution of Epac2 isoform.

**Figure 6.**
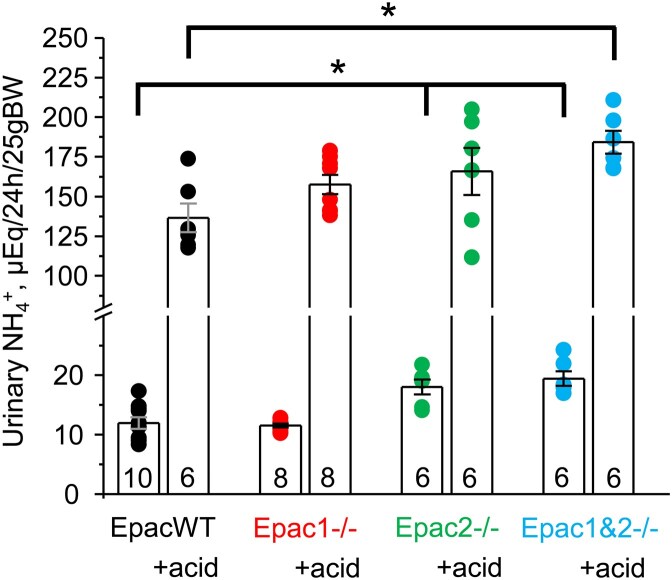
Deletion of Epac isoforms does not compromise urinary NH_4_^+^ excretion. Summary graph of 24-h urinary NH_4_^+^ levels in EpacWT (black), Epac1-/- (red), Epac2-/- (green), and Epac1&2-/- (blue) mice given 0.5% sucrose (vehicle) and 280 m m NH_4_Cl + 0.5% sucrose in drinking water for 3 d (+acid). Individual data points are shown as circles. Numbers of individual mice are shown for each group. *—significant difference (*P* < 0.05, one-way ANOVA with post-hoc Tukey test) between groups as indicated with lines and brackets on the top. Two-way ANOVA with post-hoc Tukey test shows significant (*P* < 0.05) differences when considering genotype and dietary intervention and their significant interaction (*P* = 0.02).

Ammoniagenesis is a major adaptive mechanism facilitating H^+^ excretion by the proximal tubule cells, particularly in a state of acidosis.^34^ We found at baseline, that 24-h urinary NH_4_^+^ levels were significantly elevated in Epac2-/- and Epac1&2-/- but not in Epac1-/- mice (Figure 6). We concluded that this elevation is a compensatory action for the reduced apical Na^+^/H^+^ exchange via NHE-3 in mice lacking the Epac2 isoform. Consistently, expression of the basolateral Na^+^/HCO_3_^−^ cotransporter, NBCe-1, was significantly elevated in Epac2-/- (*P* = 0.002) and Epac1&2-/- mice (*P* = 0.002), while showing only a trend (*P* = 0.30) in Epac1-/- animals (Supplementary Figure S4). Furthermore, dietary acid load produced a comparable elevation of urinary NH_4_^+^ levels in all tested groups (Figure 6), suggesting that deletion of Epac isoforms does not impair ammoniagenesis by the proximal tubule cells.

### Epac2 Deficiency Impairs H^+^ Secretion By A-type Intercalated Cells

**Figure 7.**
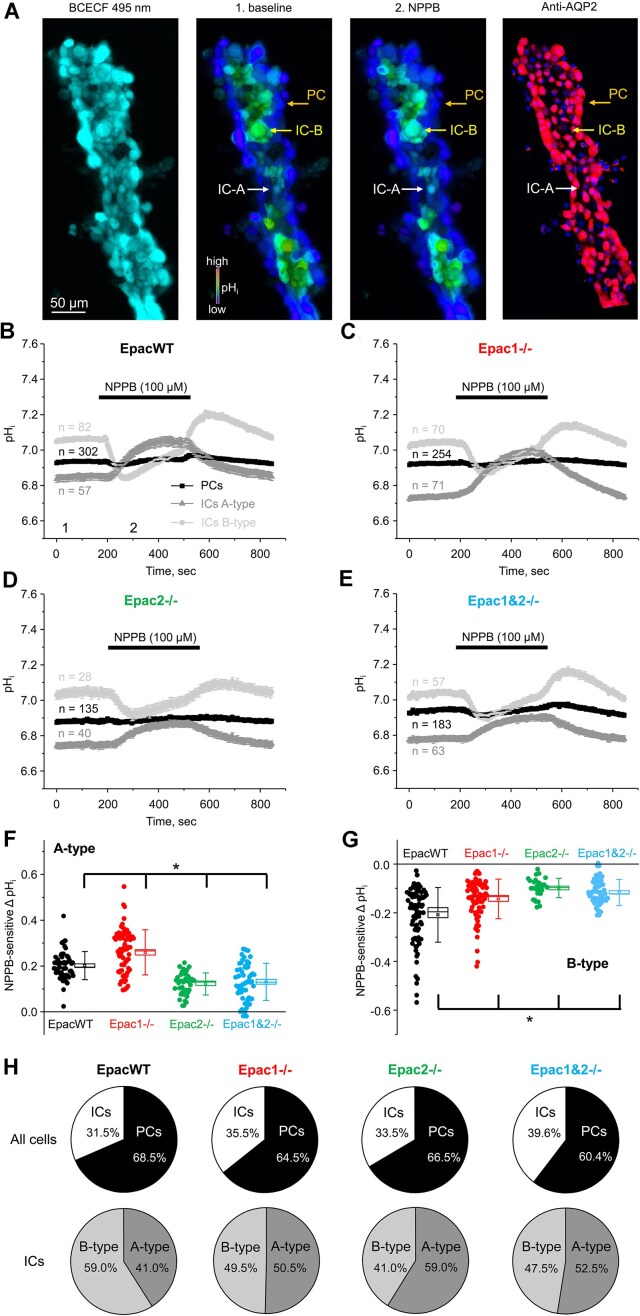
Epac2 deletion decreases Cl^−^-dependent pH_i_ changes in A-type intercalated cells of the collecting duct. (A) Representative pseudocolor images (blue—acidic and red—alkali) of intracellular pH (pH_i_) in a split-opened cortical collecting duct loaded with pH-sensitive dye BCECF at baseline (1) and after application of ClC-K2 Cl^−^ channel blocker 5-Nitro-2-(3-phenylpropylamino) benzoic acid (NPPB) to distinguish A- and B-type intercalated cells (2). A confocal micrograph of the same split-opened collecting duct probed with anti-AQP2 (pseudocolor red) to identify principal cells is shown on the right. Examples of A-type (responding with alkalization to NPPB), B-type (responding with acidification to NPPB) of intercalated cells (IC) and principal cell (PC, no change in pH_i_ to NPPB) are depicted with white, yellow, and orange arrows, respectively. Nuclear DAPI staining is shown in pseudocolor blue. The averaged time courses of changes in pH_i_ in PC (black), IC-A-type (gray) and IC-B type (light gray) in the control, upon NPPB application (shown with a black bar on top), and following washout in the collecting ducts from EpacWT (B), Epac1-/- (C), Epac2-/- (D), and Epac1&2-/- (E) mice at baseline. The number of individual tested cells for each type is indicated. Summary graphs comparing NPPB-induced changes in pH_i_ in A-type ICs (F) and B-type ICs (G) in the collecting ducts from EpacWT, Epac1-/-, Epac2-/-, and Epac1&2-/- mice. Means are shown as dots, medians are indicated by lines, bars represent standard error, and whiskers define standard deviation. *—significant change (*P* < 0.05, Wilcoxon Rank-Sum nonparametric test) versus EpacWT, as indicated with a line on the top. Three different mice and 6 individual collecting ducts were used for each experimental condition. (H) The pie charts showing ratios of ICs to PCs (top row) and A-type to B-type (bottom row) in the collecting ducts from EpacWT, Epac1-/-, Epac2-/-, and Epac1&2-/- mice.

Compromised ability to acidify urine in response to systemic acid loading in Epac knockouts (Figure 2D) indicates a defective mechanism of H^+^ secretion in the collecting duct. Thus, we next investigated the significance of Epac signaling in governing the activity of the acid-secreting A-type and base-secreting B-type of intercalated cells in freshly isolated split-opened cortical collecting ducts (Figure 7). We showed previously that application of ClC-K2 Cl^−^ channel inhibitor 5-Nitro-2-(3-phenylpropylamino) benzoic acid (NPPB, 100 µM) disrupts the Cl^−^-dependent acid-base transport in intercalated cells, which causes an increase in pH_i_ in A-type and a decrease in pH_i_ in B-type.^24^ NPPB had no effect on pH_i_ in principal cells, which were also identified by their post-experimental staining with AQP2 (Figure 7A). The averaged time courses of changes in pH_i_ in response to NPPB application for all cell types within the split-opened area of the cortical collecting ducts are shown for EpacWT (Figure 7B), Epac1-/- (Figure 7C), Epac2-/- (Figure 7D), and Epac1&2-/- (Figure 7E) mice at baseline. We next quantified the magnitudes of NPPB-induced pH_i_ changes in A- and B-type intercalated cells as indices of their overall transport rates of acids and bases. As summarized in Figure 7F, deletion of Epac1 moderately increased the Cl^−^-dependent H^+^ secretion in A-type intercalated cells when compared with EpacWT, whereas these values were significantly reduced in Epac2-/- and Epac1&2-/- mice (*P* < 0.001 for all cases). In contrast, NPPB-dependent changes in pH_i_ were significantly decreased (*P* < 0.001) in B-type intercalated cells from Epac1-/-, Epac2-/-, and Epac1&2-/- when compared with EpacWT mice (Figure 7G). We did not observe major changes in the ratio of AQP2-positive principal and AQP2-negative intercalated cells within split-opened collecting duct areas in all tested genotypes at baseline (Figure 7H). However, relatively more intercalated cells were aligned with functional A-type upon individual or concomitant Epac1 and Epac2 deletion than in EpacWT, which is indicative of a compensatory response to acidification (Figure 6H).

**Figure 8.**
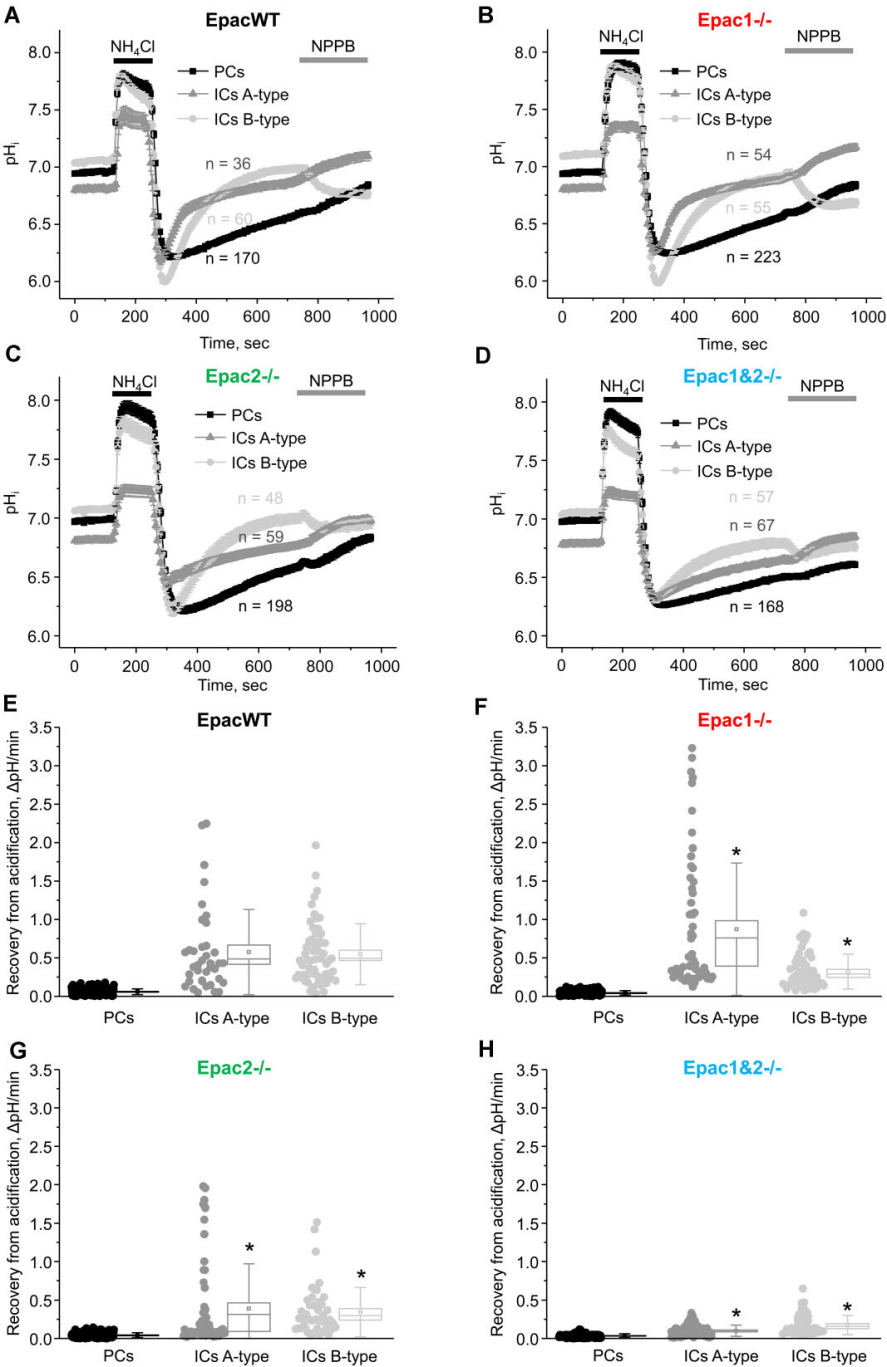
Epac2 deletion impairs H^+^ secretion in A-type intercalated cells. The summary graphs comparing the time course of pH_i_ changes in principal (PC, black), A-type (IC-A, gray), and B-type (IC-B, light gray) intercalated cells in the control, upon application of 40 m m NH_4_Cl (shown with a black bar on top), recovery after acidification, and 100 μm NPPB (shown with a gray bar on top) in the collecting ducts from EpacWT (A), Epac1-/- (B), Epac2-/- (C), and Epac1&2-/- (D) mice at baseline. The number of tested cells for each type is indicated. The respective summary graphs of recovery after acidification in PCs, ICs-A, and ICs-B types in the collecting ducts from EpacWT (E), Epac1-/- (F), Epac2-/- (G), and Epac1&2-/- (H) mice. The rate was calculated for each individual cell (shown as dots) as a linear slope of the initial pH_i_ recovery from the lowest pH_i_ value after 40 m m NH_4_Cl removal. Means are shown as dots, medians are indicated by lines, bars represent standard error, and whiskers define standard deviation. *—significant changes (*P* < 0.05, Wilcoxon Rank-Sum nonparametric test) versus the corresponding cell type in EpacWT.

We next directly examined how the deletion of Epac isoforms affects the rates of pH_i_ recovery after acidification, as an index of H^+^ extrusion, in split-opened cortical collecting ducts (Figure 8). We also applied an inhibitor of the basolateral Cl^−^ conductance, NPPB (100 µm), to discriminate the signals from A- and B-types of intercalated cells following the intracellular acidification-recovery protocol. Figure 8 panels A-D show the averaged time courses of pH_i_ changes for each cell type for EpacWT, Epac1-/-, Epac2-/-, and Epac1&2-/- mice at baseline. The respective summary graphs for each individual cell type are shown in panels E-H. There were no differences in the recovery rates in principal cells between all tested strains, as expected. Deletion of Epac1 significantly accelerated the recovery from acidification in A-type intercalated cells compared with the values seen in EpacWT, indicative of an augmented H^+^ secretion (Figure 8F). In contrast, these recovery rates were significantly reduced in A-type intercalated cells from Epac2-/- (Figure 8G) and Epac1&2-/- (Figure 8H) mice (*P* < 0.001). We noted that the initial NH_4_Cl pulse induces a lower level of alkalization in A-type intercalated cells of Epac2-/- and Epac1&2-/-, when compared to EpacWT mice (Figure 8A, C, D). However, this is unlikely to represent an altered intracellular buffering capacity, which is primarily determined by the initial slope of pH_i_ changes (ie, a steeper slope would correspond to a reduced capacity). Instead, we assume that this is a result of altered activity/expression of H^+^-contributing transporting systems in A-type intercalated cells of the knockouts. We next found that the recovery rates were significantly decreased in B-type intercalated cells from Epac1-/-, Epac2-/-, and Epac1&2-/- mice when compared with EpacWT (*P* < 0.001). Consistently, Western blot analysis revealed a significantly lower (*P* < 0.005) expression of the apical HCO_3_^−^/Cl^−^ exchanger, pendrin, in Epac-isoform deficient mice (Supplementary Figure S5). While a reduction in pendrin expression is anticipated in B-type intercalated cells of Epac knockout mice, it might also indicate a deficient regulation of base transport. However, it is unlikely that this contributes significantly to the observed metabolic acidosis, particularly in Epac2-/- and Epac1&2-/- mice.

### Epac2 Deficiency Decreases V-ATPase and AE-1 Expression and Membrane Localization in the Collecting Duct to Reduce Excretion of Titratable Acids in Urine

**Figure 9.**
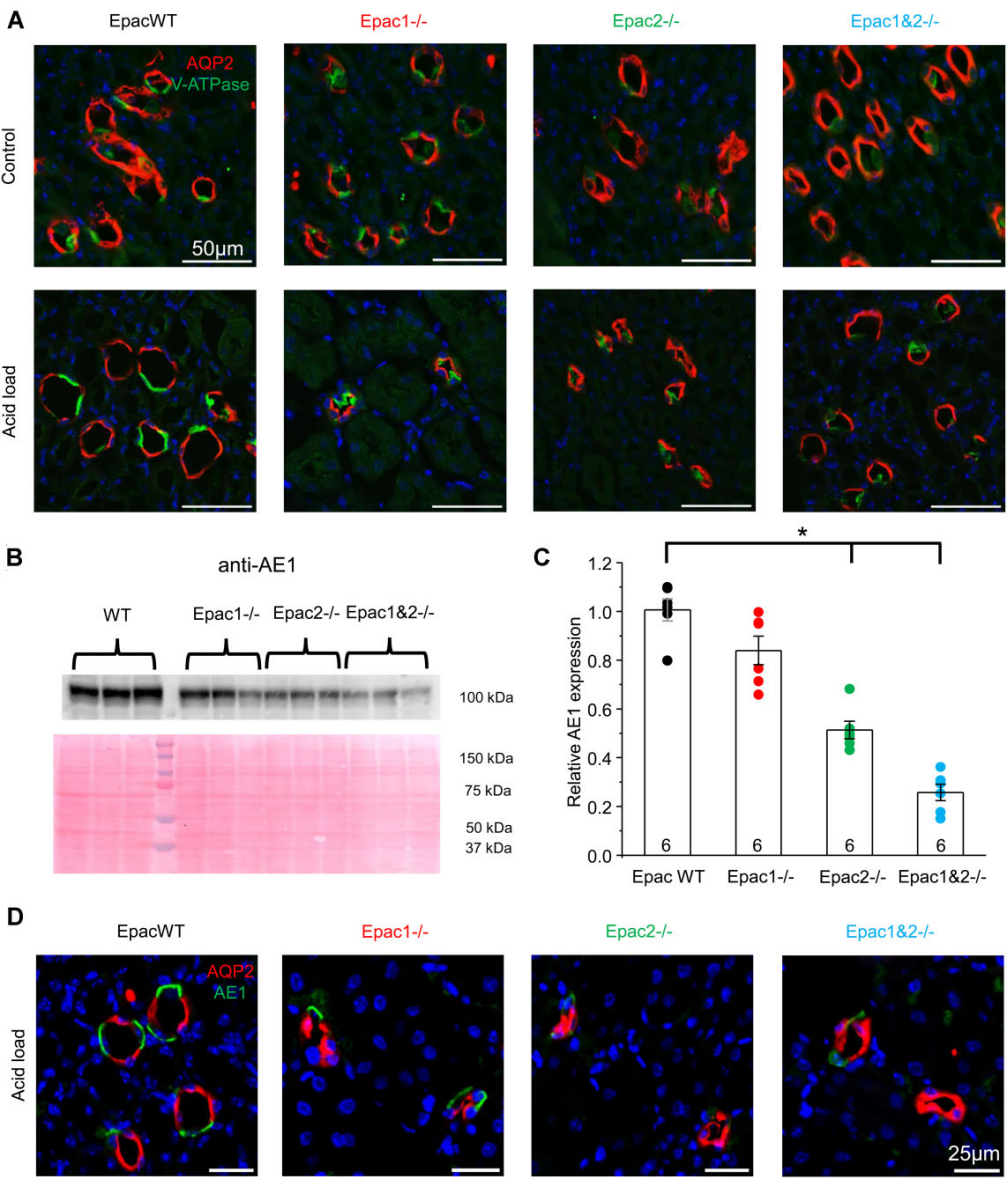
Deletion of Epac2 compromises translocation and expression of acid-base transporters in A-type intercalated cells. (A) Representative confocal images of kidney sections probed with antibodies against the a4 subunit of V-ATPase (pseudocolor green) and anti-AQP2 (pseudocolor red) in EpacWT, Epac1-/-, Epac2-/-, and Epac1&2-/- mice given 0.5% sucrose vehicle (control) and 280 m m NH_4_Cl + 0.5% sucrose in drinking water for 3 d (acid load). All images were taken with identical laser intensity settings for each wavelength. Nuclear DAPI staining is shown in pseudocolor blue. (B) Representative Western blot probed with anti-AE1 antibodies from whole kidney lysates of EpacWT, Epac1-/-, Epac2-/-, and Epac1&2-/- mice at baseline, as indicated with brackets on the top. Each lane represents an individual animal. Ponceau red staining of the same nitrocellulose membrane is shown below to demonstrate equal protein loading. (C) Summary graph comparing AE1 expression from the Western blots similar to those shown in panel B. The intensity values were normalized to the total signal of the respective lanes in Ponceau red staining. *—significant decrease (*P* < 0.05; one-way ANOVA with post-hoc Tukey test) versus EpacWT, as indicated with a bracket on the top. (D) Representative confocal images of kidney sections probed with anti-AE1 (pseudocolor green) and anti-AQP2 (pseudocolor red) in EpacWT, Epac1-/-, Epac2-/-, and Epac1&2-/- mice given 280 m m NH_4_Cl + 0.5% sucrose in drinking water for 3 d (acid load). Low magnification images of AE1 and AQP2 expression in WT and Epac isoform knockouts in the control and after dietary acidification are shown in Supplementary Figure S7. All images were taken with identical laser intensity settings for each wavelength. Nuclear DAPI staining is shown in pseudocolor blue.

Previous evidence demonstrated a critical role of cAMP-dependent signaling for the translocation of the V-ATPase pump to the apical membrane in the collecting duct A-type intercalated cells to increase H^+^ secretion.^35^ Therefore, we tested how deletion of Epac isoforms affects V-ATPase subcellular localization using immunofluorescent confocal microscopy. As shown in representative images of kidney sections in Figure 9, a4-subunit V-ATPase-reporting signal was most prominent in the apical regions of AQP2-negative intercalated cells of EpacWT and Epac1-/- mice at baseline, whereas the diffuse cytosolic staining was observed in Epac2-/- and Epac1&2-/- mice. Dietary acidification further increased the apical accumulation of V-ATPase fluorescent signal in EpacWT and Epac1-/- mice (Figure 9A, lower panel). In contrast, the apical translocation of V-ATPase was largely absent in Epac2-/- and Epac1&2-/- mice. The expression of a4-subunit V-ATPase was also detected in the proximal tubule brush border (Supplementary Figure S6), which is consistent with previous reports.^36^ However, the intensity of the fluorescent signal was much weaker when compared with that in the collecting duct intercalated cells. Thus, we did not follow this further.

We next investigated whether the impaired subcellular localization and apical trafficking of the V-ATPase H^+^ pump upon Epac2 deficiency are also accompanied by compromised function of the basolateral anion exchanger 1 (AE1 or Slc4A1), which is specific for A-type. Immunofluorescent analysis of AE1 expression using confocal microscopy revealed similar localization of the AE1-reporting fluorescent signal to the basolateral membrane in EpacWT and Epac isoform-deficient mice at baseline (Supplementary Figure S7). However, we detected much weaker signal intensity under the same laser intensity settings in Epac2-/- and Epac1&2-/-mice. Consistently, Western blot analysis (Figure 9B, C) revealed that deletion of Epac2 and Epac1&2 but not Epac1 significantly (*P* < 0.001) decreased AE1 levels when compared with EpacWT mice. Moreover, dietary acidification induced a marked upregulation of the AE1 fluorescent signal at the apical region in EpacWT and Epac1-/-, but not in Epac2-/- and Epac1&2-/-mice (Figure 9D and Supplementary Figure S7). Taken together, our results demonstrate that deletion of Epac2, but not Epac1, compromises H^+^ secretion in A-type intercalated cells by disrupting the apical translocation of V-ATPase and reducing the expression of the basolateral AE1 levels.

**Figure 10.**
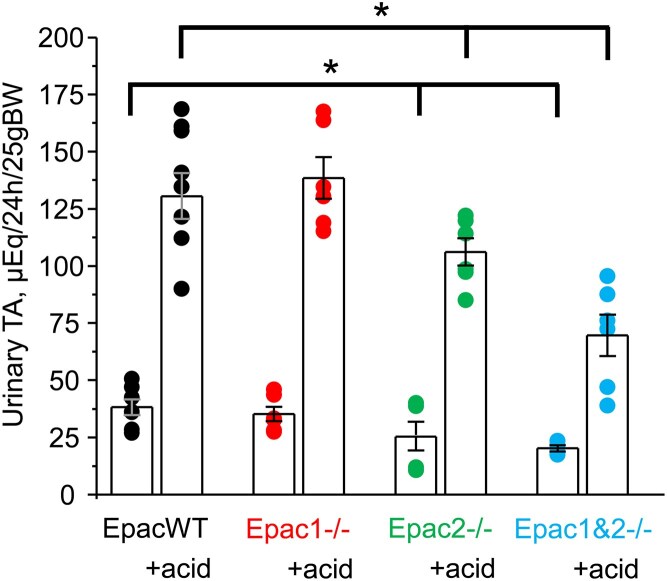
Deletion of Epac2, but not Epac1, decreases urinary excretion of titratable acids (TA). Summary graph of 24-h urinary TA levels in EpacWT (black), Epac1-/- (red), Epac2-/- (green), and Epac1&2-/- (blue) mice given 0.5% sucrose vehicle and 280 m m NH_4_Cl + 0.5% sucrose in drinking water for 3 d (+acid). Individual data points are shown as circles. The number of individual mice is shown for each group. *—significant difference (*P* < 0.05, one-way ANOVA with post-hoc Tukey test) between groups as indicated with lines and brackets on the top. Two-way ANOVA with post-hoc Tukey test shows significant (*P* < 0.05) differences when considering genotype and dietary intervention and their significant interaction (*P* = 0.007).

Finally, we inquired how deletion of Epac isoforms affected excretion of titratable acids (TA) in urine. As shown in the summary graph (Figure 10), there were no differences (*P* = 0.53) in the 24-h TA levels between EpacWT and Epac1-/- mice. In contrast, the baseline TA levels were significantly decreased in Epac2-/- (*P* = 0.03) and Epac1&2-/- (*P* = 0.004) mice. Dietary acidification led to comparable elevations in TA levels in EpacWT and Epac1-/- mice, but these increases were significantly less pronounced in Epac2-/- (*P* = 0.03) and Epac1&2-/- (*P* < 0.001) mice.

In summary, our results suggest a critical role of the Epac2 isoform in regulating H^+^ secretion in A-type intercalated cells of the collecting duct under both basal and acidotic conditions. Epac2 deficiency leads to impaired TA excretion and, subsequently, the inability to properly acidify urine. In contrast, Epac1 contribution is largely dispensable.

## Discussion

**Figure 11.**
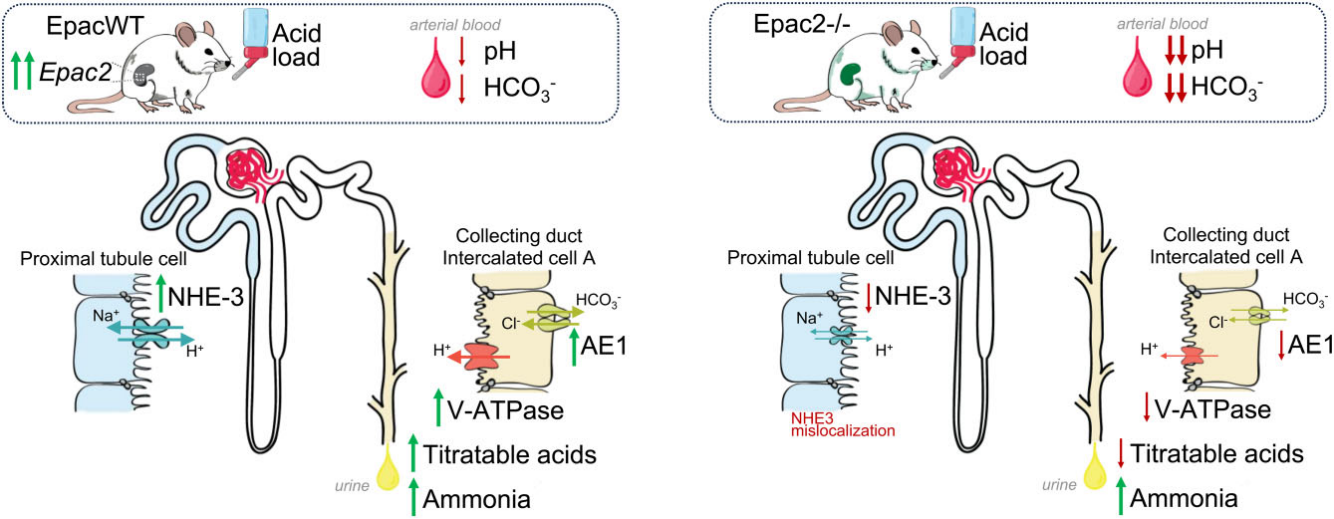
Principal scheme of contribution of Epac2 isoform in renal adaptation to dietary acid load. Epac2–exchange protein directly activated by cAMP isoform 2. NHE-3–Na^+^/H^+^ exchanger type 3 (*Slc9a3*); V- ATPase—vacuolar-type ATPase H^+^ pump, AE1–anion exchanger type 1 (*Slc4a1*).

In this study, we uncovered a novel physiologically relevant role of the Epac signaling cascade and particularly Epac2 isoform in restoring acid-base balance after dietary acidification. Deletion of Epac2 compromises H^+^ transport along the renal nephron (Figure 11). First, it decreases the apical Na^+^/H^+^ exchange in the proximal tubule by reducing NHE-3 levels and targeting the transporter toward the base of the brush border, where it is less active. Second, it impedes H^+^ secretion by A-type intercalated cells of the collecting duct in part by impairing the apical translocation of V-ATPase and by decreasing expression of the AE1 at the basolateral membrane. Epac2-/- mice are able to maintain physiological pH at the baseline. However, dietary acidification unmasks the deficient H^+^ secretion, resulting in severe metabolic acidosis due to the inability to acidify urine. The regulation of systemic acid-base balance by the Epac signaling cascade appears to be isoform-specific, since deletion of Epac1 has a considerably weaker effect on NHE-3 levels and does not affect H^+^ secretion in the collecting duct. Epac1-/- mice have normal urinary pH at baseline and are able to decrease it in response to dietary acidification exhibiting a comparable adaptation with EpacWT. It should be noted that male mice were exclusively used to generate experimental data. This means that the extent of systemic acid-base balance distortions, as well as the H^+^ transport rates in the proximal tubule and the collecting duct, might differ in females lacking Epac2 isoform. Interestingly, notable sex-dependent differences in major transporting systems along the renal tubule have been reported.^37^ With respect to this topic, females have less NHE-3-dependent Na^+^ reabsorption and subsequently less HCO_3_^−^ uptake in the proximal tubule. Thus, it is reasonable to assume that deletion of Epac2 might result in even stronger metabolic acidosis upon dietary acidification in females than in males. However, this is a subject for future direct testing.

Previous studies have shown that the kidney has one of the highest levels of Epac1 expression among different tissues; in contrast, Epac2 expression there is considerably lower, with the highest levels being observed in the central nervous system, pancreas, and the adrenal gland.^8^ Despite this, we found a much greater contribution of the less abundant Epac2 than the more abundant Epac1 in the regulation of renal acid-base handling (Figure 2). In fact, the acidotic phenotype of Epac1&2-/- is only marginally worse than that observed in Epac2-/- mice (Figure 2). However, the effects of Epac1 do not seem to be dispensable, at least in the proximal tubule, where concomitant deletion of Epac1 and Epac2 has additive effects on decreasing NHE-3 expression, activity, and localization to the tip of the brush border (Figure 5 and Supplementary Figure S3). At the same time, Epac2 deficiency also compromises H^+^ transport by the A-type intercalated cells of the collecting duct and subsequently decreases titratable acid excretion in urine (Figures 7-10). On the contrary, we found that Epac1-/- mice have V-ATPase localization to the apical membrane and AE1 expression at the basolateral membrane in A-type intercalated cells, which were comparable with those in EpacWT (Figure 9). Moreover, our studies in freshly isolated split-opened cortical collecting ducts revealed an augmented Cl^−^-dependent H^+^ transport (Figure 7) and accelerated recovery after intracellular acidification (Figure 8) in A-type intercalated cells from Epac1-/- mice. This was further accompanied by a reduction of pendrin levels in B-type intercalated cells (Supplementary Figure S5). Of note, a similar downregulation of pendrin and upregulation of V-ATPase B1 subunit were reported in mice lacking NHE-3 in the proximal tubule to limit the loss of HCO_3_^−^ in the proximal tubule.^38^ This indicates that the intact regulation of acid-base transport in the collecting duct of Epac1-/- mice explains the much weaker metabolic acidosis in response to dietary acid loading (Figure 2). It should be noted, though, that Epac2 deficiency might also lead to defective regulation of base transport in B-type intercalated cells, despite a similar compensatory downregulation of pendrin expression in Epac1-/- and Epac2-/- mice. Indeed, Epac2 and Epac1&2 knockouts have lower urinary pH at the baseline (Figure 2) despite defective H^+^ transport in both the proximal tubule and collecting duct. This seemingly perplexing phenotype could be explained by additional disruption of pendrin-mediated HCO_3_^−^ secretion. This possibility should be explored in a separate study.

Previously published evidence suggests similar but not overlapping expression of both Epac isoforms in different segments of the renal nephron, with the exception of the collecting duct.^21^ It is quite puzzling, however, that Epac1 expression was reported to be higher in intercalated cells, whereas Epac2 presence was more prominent in principal cells of the collecting duct.^21^ It is possible that the Epac1 isoform has more secondary roles or is redundant to Epac2 with respect to controlling the transport of acids and bases. Interestingly, we reported previously that deletion of either Epac isoform decreases ENaC-mediated Na^+^ reabsorption in the principal cells to a similar extent. There was no further decrease in ENaC activity upon concomitant deletion of Epac isoforms.^23^ This indicates that the Epac cascade has multifaceted roles in the collecting duct, with both isoforms being indispensable for Na^+^ handling. In contrast, only Epac2 contributes to acid-base homeostasis.

One of the important observations of this study is that renal Epac2 expression is upregulated upon dietary acid load, while Epac1 levels remain virtually the same (Figure 3). This is consistent with the notion that Epac2 is a critical component of the adaptor pH-sensing signaling machinery, which facilitates the excretion of acidic load in urine to maintain acid-base balance. Indeed, we found that Epac2 deletion produces a nearly normal phenotype at baseline, with the single exception of a lower urinary pH. In contrast, Epac2-/- mice develop a severe hyperchloremic (non-gap) metabolic acidosis upon dietary acidification (Figure 2). It is reasonable to propose that the augmented renal Epac2 expression is critical for upregulating Na^+^/H^+^ exchange via NHE-3 in the proximal tubule and for stimulating H^+^ secretion by promoting V-ATPase targeting to the apical membrane and augmenting expression of the basolateral AE1 in the A-type of intercalated cells (Figures 9–10). In recent years, a special subclass of G-protein coupled receptors (GPCRs) termed “pH receptors” was uncovered. This includes G-protein receptor 4 (GPR4), T cell death-associated gene 8 (TDAC8, or GPR65), and ovarian cancer G-protein coupled receptor 1 (OGR1, or GPR68). All these receptors can be directly activated by physiologically relevant decreases in pH (in the 7.5-6.5 range).^39,40^ GPR4 and OGR1 have been found in the kidney, specifically in the collecting duct and the proximal tubule/glomerulus, respectively.^41,42^ Intriguingly, activation of GPR4 leads to an elevation of cAMP levels indicative of Epac activation.^39^ GPR4-/- mice develop hyperchloremic (non-gap) metabolic acidosis due to decreased net acid secretion by the kidney.^42^ Furthermore, mRNA levels of AE1 were markedly lower in acid-loaded GPR4-/- than in GPR4+/+ mice.^43^ The apparent similarities between defective acid-base handling in Epac2-/-, Epac1&2-/- (Figures 2, 9) and GPR4-/- mice indicate that Epac2 could be a critical downstream effector of GPR4 in the collecting duct to regulate H^+^ secretion by intercalated cells. In contrast, Epac1 deletion does not interfere with H^+^ secretion in the collecting duct (Figures 7-8) and thus does not seem to be downstream of GPR4 activation. This hypothesis needs to be directly tested in future studies. Consistently, previous studies have shown that activation of GPR4 by low pH regulates adhesion of human umbilical vein endothelial cells (HUVECs) mainly through the Gs/cAMP/Epac pathway.^44^ In contrast to GPR4, activation of OGR1 does not seem to contribute to the regulation of acid-base balance, while it is responsible for the enhanced urinary Ca^2+^ excretion during acidosis.^41^ In fact, it was found that NHE-3 levels in the proximal tubule were elevated in OGR1-/- mice upon dietary acidification.^41^ Considering that OGR1 activation leads to inositol-triphosphate (IP_3_) production and elevated [Ca^2+^]_i_,^39^ it is unlikely that Epac2 contributes to this pathway.

We show that the impaired Epac2 function in Epac2-/- and Epac1&2-/- mice exhibits a defect in acid-base homeostasis reminiscent of type I renal tubule acidosis (RTA), characterized by the inability to acidify urine during metabolic acidosis.^3^ RTA type I has the distal nephron origin (for instance, loss-of-function mutations in AE1 or β1/α4 subunits of V-type ATPase), which is consistent with mis-localized V-ATPase (Figure 9A), reduced AE1 expression (Figure 9B, C), impaired H^+^ secretion (Figures 7, 8), and reduced titratable acid excretion, when compared to EpacWT (Figure 10) observed in both knockouts. It is quite unexpected that Epac2-/- and Epac1&2-/- mice are able to increase titratable acid excretion (Figure 10) without a notable decrease in the urinary pH after dietary acidification (Figure 2D). This may be related to changes in the excretion rates of other physiologically relevant buffers, such as creatinine and phosphate. In fact, a significant reduction in proximal tubule NHE-3 levels (Figure 4A, B) indicates a decreased glomerular filtration rate (GFR) and, consequently, urinary excretion of creatinine in Epac-isoform knockouts at baseline. Dietary acidification increases NHE-3 abundance (Figure 4C, D), thereby causing a partial restoration of GFR and enhancing creatinine excretion in the urine. This, in turn, would mitigate urinary acidification despite the increased titratable acid excretion in Epac2-/- and Epac1&2-/- mice. While a complementary contribution of potential changes in urinary phosphate could not be discarded, the previous studies did not find a role for Epac signaling in regulating the proximal tubule Na^+^-phosphate cotransporter (NaPi-IIa, *Slc34A1*).^45,46^

We also found that Epac2 deficiency causes decreased NHE-3 expression, localization, and H^+^ secretion in the proximal tubule. Impaired acid-base transport in the proximal tubule is a hallmark of type II (proximal) RTA.^3^ It is worth noting that NHE-3 dysfunction alone neither causes type II RTA nor results in notable alterations in the systemic acid-base balance. While global NHE-3 deletion produces a severe metabolic acidosis in mice, this was attributed to its function in the intestinal epithelia, where its deletion causes distended abdomens, fluid-filled abdominal loops, and alkaline diarrhea.^47^ Neither Epac isoform knockout exhibited gastrointestinal problems and had acidic pH at baseline, arguing that Epac signaling does not contribute to the regulation of NHE-3 in intestinal epithelia (Figure 2). Interestingly, NHE-3 deletion either in the kidney or specifically in the proximal tubule did not produce major defects in acid-base balance at baseline (reviewed in^48^). Our results demonstrate that deletion of Epac1 or Epac2 causes a reduction in renal NHE-3 expression, localization, and activity (Figures 4-5). Moreover, this regulation seems to be additive, at least at the level of NHE-3 expression (Figure 4A, B). The decreased NHE-3 function is likely causing a marginally stronger metabolic acidosis in Epac1-/- than in EpacWT mice after dietary acidification (Figure 2). Ammoniagenesis is a powerful mechanism facilitating acid excretion as NH_4_^+^ and the generation of new HCO_3_^−^, predominantly from glutamine in acidotic states.^34^ Despite decreased NHE-3 expression and activity, we found that urinary excretion of ammonium was not compromised in Epac isoform knockouts (Figure 6). This suggests that NHE-3 is not a limiting factor for NH_3_/NH_4_^+^ secretion into the tubular lumen in the knockouts. While NH_4_^+^ can substitute H^+^ in the NHE-3 dependent Na^+^/H^+^ exchange, other mechanisms, including direct diffusion of NH_3_ across the plasma membrane and NH_4_^+^ movement across K^+^ channels in the proximal tubule, could also contribute.^34^ Interestingly, deletion of the proximal tubule K_ir_4.2 potassium channel caused hyperchloremic metabolic acidosis, in part, due to defective ammonia metabolism and excretion.^49^ While we did not specifically test the expression of major enzymes involved in the generation of ammonia from glutamate, such as GDH and PEPCK, we did not detect any signs that deletion of Epac isoforms compromises ammoniagenesis. In fact, urinary NH_4_^+^ levels were modestly elevated in Epac2-/- and Epac1&2-/- mice (Figure 6). Moreover, expression of the basolateral Na^+^/HCO_3_^−^ cotransporter, NBCe1, was upregulated (Supplementary Figure S4), which is indicative of the augmented compensatory ammoniagenesis in mice with deleted Epac2 isoform.

In summary, our study provides the first direct evidence of the significance of Epac signaling in the kidney for handling dietary acid load, with a critical contribution of the Epac2 isoform. We unravel the mechanistic aspects of the severe metabolic acidosis in mice lacking Epac2, which involves impaired H^+^ secretion in both the proximal and distal segments of the renal nephron, resulting in the inability to acidify urine in response to dietary acid load. Importantly, the overall gene ontology analysis comparing EpacWT and Epac1&2-/- mice demonstrates a dominant association with acid-base disturbances and specifically metabolic acidosis, from the perspective of the human phenotype (Figure 1). Dietary acid load is a very common nutritional and social phenomenon, considering the consumption of processed foods, high amounts of animal protein, and elevated salt consumption.^1^ Inadequate elimination of dietary acids by the kidney can lead to a chronic metabolic acidosis, which if left untreated, is a risk factor for the development of cardiovascular diseases, metabolic syndrome/insulin resistance, and cancer, thereby significantly increasing morbidity and mortality rates.^1,3,50,51^ In fact, nearly half of the patients admitted to the emergency room present with a perturbed acid-base balance.^52,53^ Here, we demonstrate that upregulation of Epac2 in the kidney is imperative for proper physiological response to dietary acidification. While there are no available FDA-approved drugs targeting the Epac signaling pathway, our study brings attention to the possibility of regulating acid-base balance with the future development of Epac2-targeting pharmacological tools.

## Supplementary Material

zqaf048_Supplemental_Files

## Data Availability

This study does not involve the development of custom software/code, models, algorithms, protocols, or methods. The data that support the findings of this study are available in the Materials and Methods, Results, and/or Supplemental Material of this article.
