# Supplementary material for: Epac2 Deficiency Compromises Adaptation to Dietary Acidification by Decreasing H+ Transport in the Renal Nephron
Source: Function (Oxf). 2025 Oct 14;6(6):zqaf048. doi: 10.1093/function/zqaf048 (PMC12586993; doi:10.1093/function/zqaf048)

## SUPPLEMENTARY FIGURES

**Supplementary Figure 1. Comparable water intake and urine output in Epac isoform-deficient mice subjected to dietary acid load.** Summary graphs of 24-hour water intake **(A)** and urinary volume **(B)** in EpacWT (black), Epac1-/- (red), Epac2-/- (green), and Epac1&2-/- (blue) mice. Animals were given 0.5% sucrose vehicle at day 0, followed by dietary acidification with 280 mM $NH_4Cl$ + 0.5% sucrose in drinking water for 3 days (+acid). Consecutive measurements from the same animal are connected by lines. * - significant difference ($p < 0.05$, one-way ANOVA with post-hoc Tukey test) between groups as indicated with lines and brackets on the top.

**Supplementary Figure 2. Dietary acidification causes mild hypernatremia and normokalemia in WT and Epac isoform-deficient mice.** Summary graphs of arterial $Na^+$ **(A)** and $K^+$ **(B)** levels in EpacWT (black), Epac1-/- (red), Epac2-/- (green), and Epac1&2-/- (blue) mice given 0.5% sucrose (vehicle) and with 280 mM $NH_4Cl$ + 0.5% sucrose in drinking water for 3 days (+acid). Individual values are shown as circles. Numbers of individual mice are shown for each group. * - significant difference ($p < 0.05$, one-way ANOVA with post-hoc Tukey test) between groups as indicated with lines and brackets on the top.

**Supplementary Figure 3. Improved spatial resolution and signal-to-noise ratio with STED super-resolution microscopy to demonstrate NHE-3 mis-localization along the brush border in Epac-deficient mice.** **(A)** Representative images of NHE-3 fluorescent signal in individual proximal tubules captured with regular confocal (left) and STED (middle) microscopy. An overlay of both images is shown in the right panel. Pseudocolor represents the intensity of fluorescent signal (violet – low, yellow - high). **(B)** Low magnification image of the same proximal tubule within a renal section, with NHE-3 expression shown in pseudocolor green. **(C)** Comparison of relative fluorescent intensity along the white dashed line in panel A, taken with confocal (black) and STED (red) microscopy. The signals were normalized to their respective maximal values. **(D)** Representative STED images of individual proximal tubules probed with antibodies against NHE-3 (pseudocolor green, top row), brush border marker villin (pseudocolor red, middle row), and the respective merged images (bottom row) from EpacWT, Epac1-/-, Epac2-/-, and Epac1&2-/- mice given 280 mM $NH_4Cl$ + 0.5% sucrose in drinking water for 3 days.

**Supplementary Figure 4. Increased Na/HCO3- cotransporter NBCe1 levels in mice lacking Epac2 isoform. (A)** Representative Western blot probed with anti-NBCe1 antibodies from whole kidney lysates of EpacWT, Epac1-/-, Epac2-/-, and Epac1&2-/- mice at baseline, as indicated with brackets on the top. Each lane represents an individual animal. Ponceau red staining of the same nitrocellulose membrane is shown below to demonstrate equal protein loading. **(B)** Summary graph comparing NBCe1 expression from the Western blots similar to those shown in panel A. The intensity values were normalized to the total signal of the respective lanes in Ponceau red staining. * - significant decrease ($P < 0.05$; one-way ANOVA with post-hoc Tukey test) versus EpacWT, as indicated with a bracket on the top.

**Supplementary Figure 5. Mice lacking the Epac1 or Epac2 isoform have decreased pendrin levels. (A)** Representative Western blot probed with anti-pendrin antibodies from whole kidney lysates of EpacWT, Epac1-/-, Epac2-/-, and Epac1&2-/- mice at baseline, as indicated with brackets on the top. Each lane represents an individual animal. Ponceau red staining of the same nitrocellulose membrane is shown below to demonstrate equal protein loading. **(B)** Summary graph comparing pendrin expression from the Western blots similar to those shown in panel A. The intensity values were normalized to the total signal of the respective lanes in Ponceau red staining. * - significant decrease ($P < 0.05$; one-way ANOVA with post-hoc Tukey test) versus EpacWT, as indicated with a bracket on the top.

**Supplementary Figure 6. Segment-specific expression of the a4 subunit of V-ATPase in mouse kidney.** Representative confocal image from a kidney section probed with anti-a4 subunit of V-ATPase (pseudocolor green) and anti-AQP2 (pseudocolor red) in EpacWT mouse. Strong V-ATPase-reporting signal is present in AQP2-positive collecting ducts (CD), whereas a weak staining is observed in the brush border area of proximal tubules (PT). Nuclear DAPI staining is shown in pseudocolor blue.

**Supplementary Figure 7. Decreased expression and impaired adaptation of AE1 to dietary acidification in mice lacking Epac2 isoform.** Representative confocal images of kidney sections probed with anti-AE1 (pseudocolor green) and AQP2 (pseudocolor red) in EpacWT, Epac1-/-, Epac2-/-, and Epac1&2-/- mice given 0.5% sucrose vehicle (control) and 280 mM $NH_4Cl$ + 0.5%

sucrose in drinking water for 3 days (acid load). All images were taken with identical laser intensity settings for each wavelength. Nuclear DAPI staining is shown in pseudocolor blue.

Supplemental Figure 1

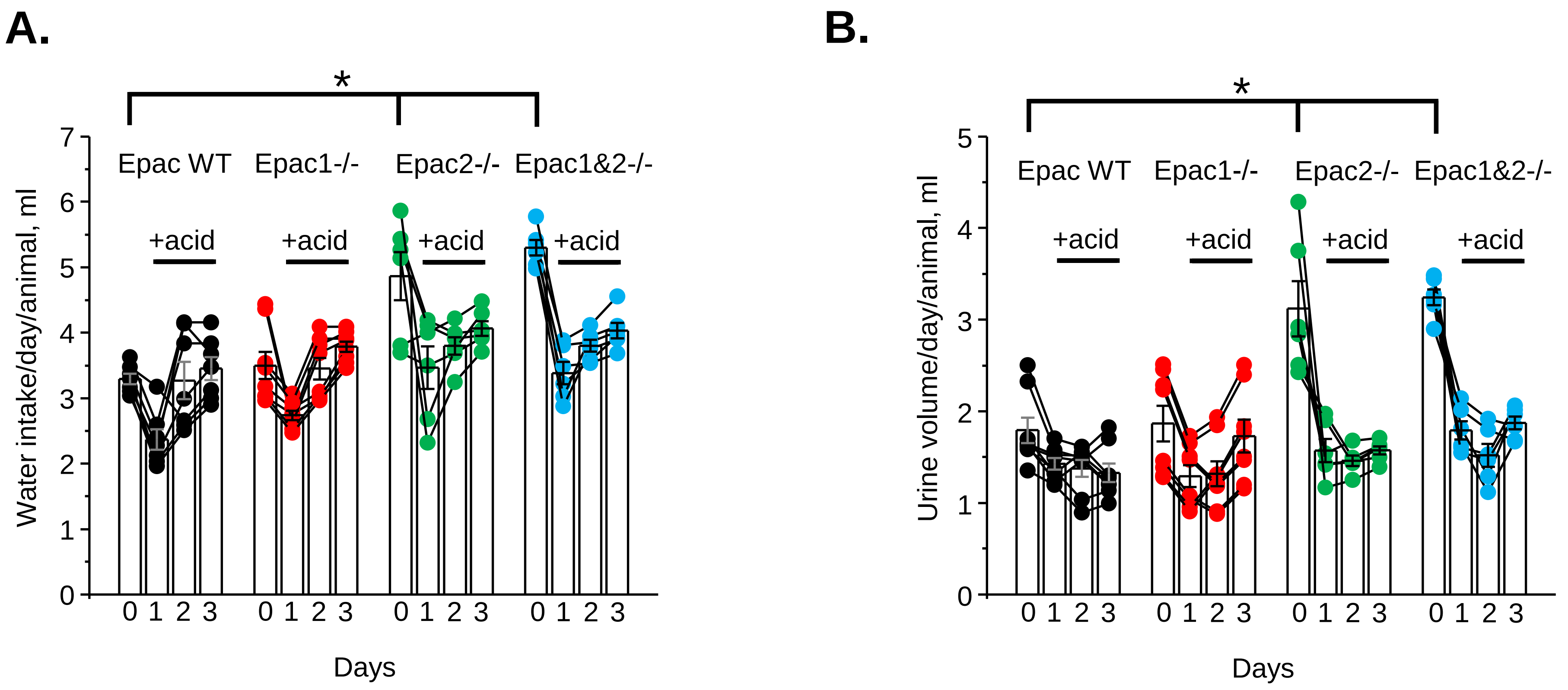

Supplemental Figure 2

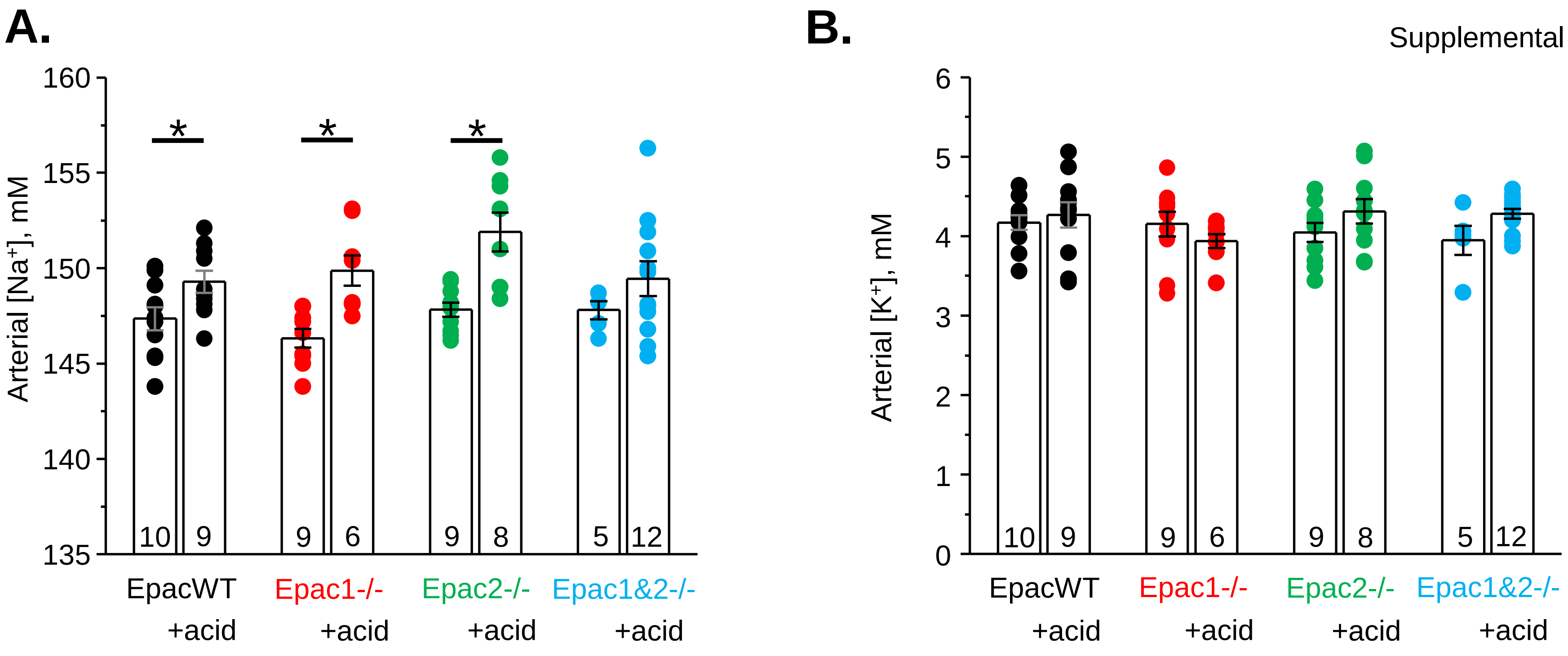

Supplemental Figure 3

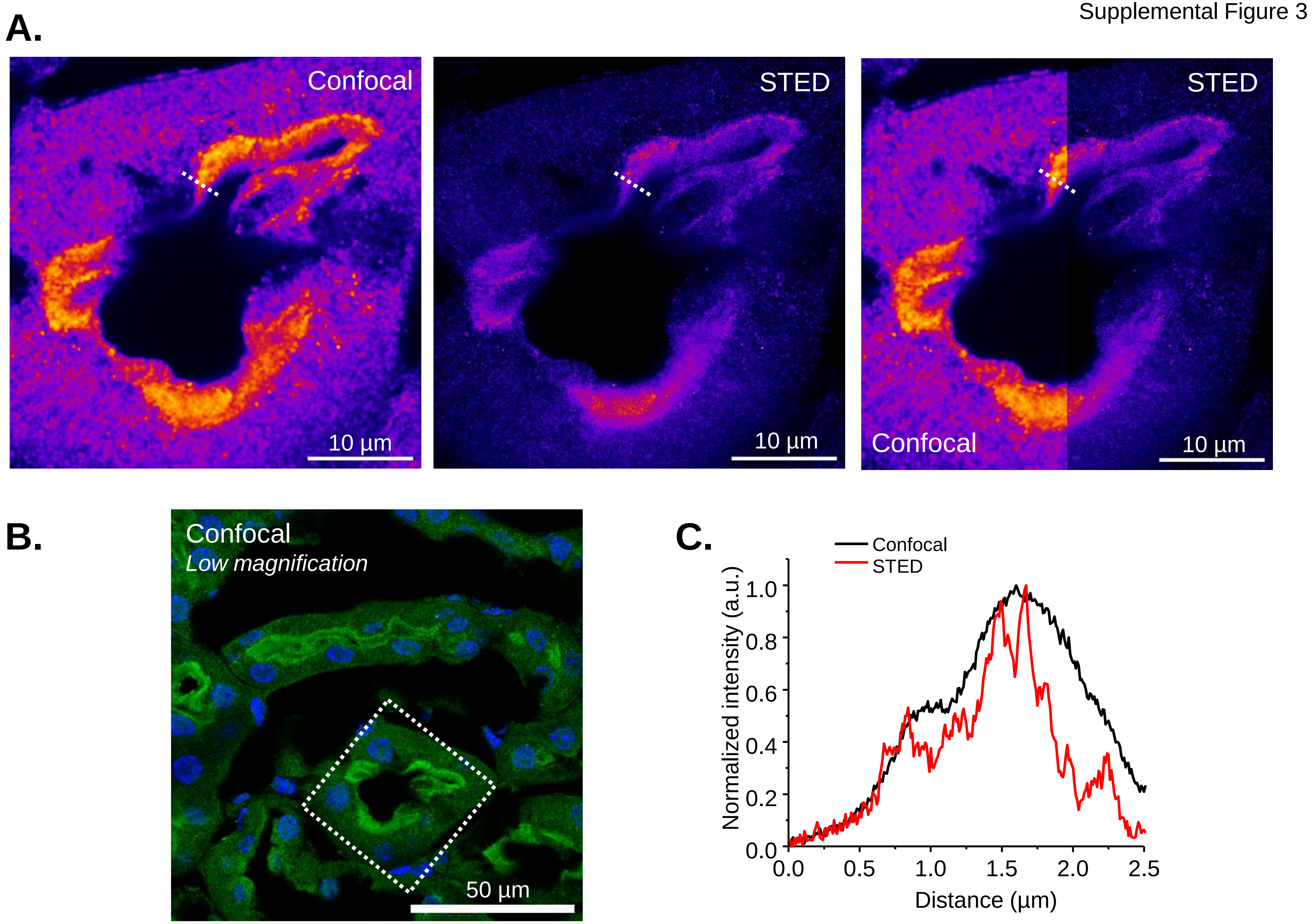

Supplemental Figure 3

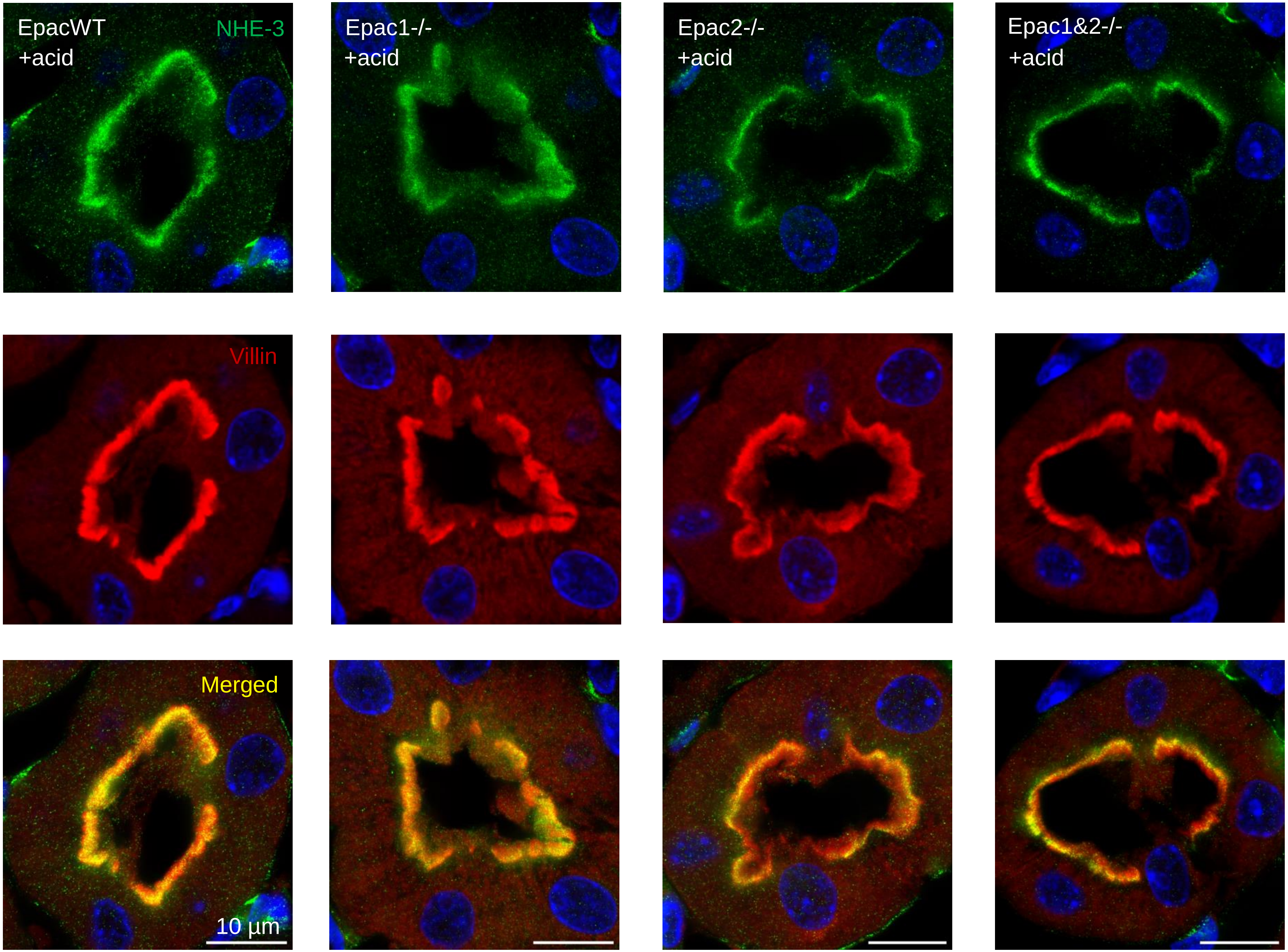

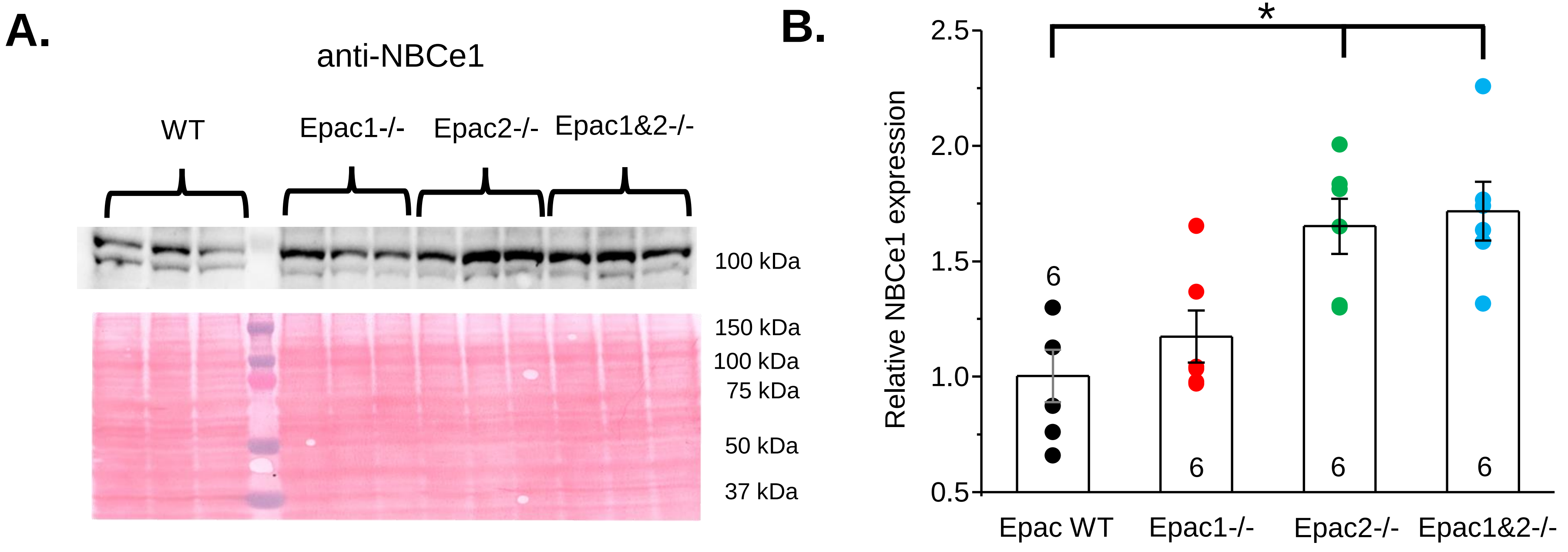
A.
anti-NBCe1
WT
Epac1-/-
Epac2-/-
Epac1&2-/-
100 kDa
150 kDa
100 kDa
75 kDa
50 kDa
37 kDa
B.
*
2.5
2.0
1.5
1.0
0.5
Relative NBCe1 expression
6
6
6
6
Epac WT
Epac1-/-
Epac2-/-
Epac1&2-/-

Supplemental Figure 5

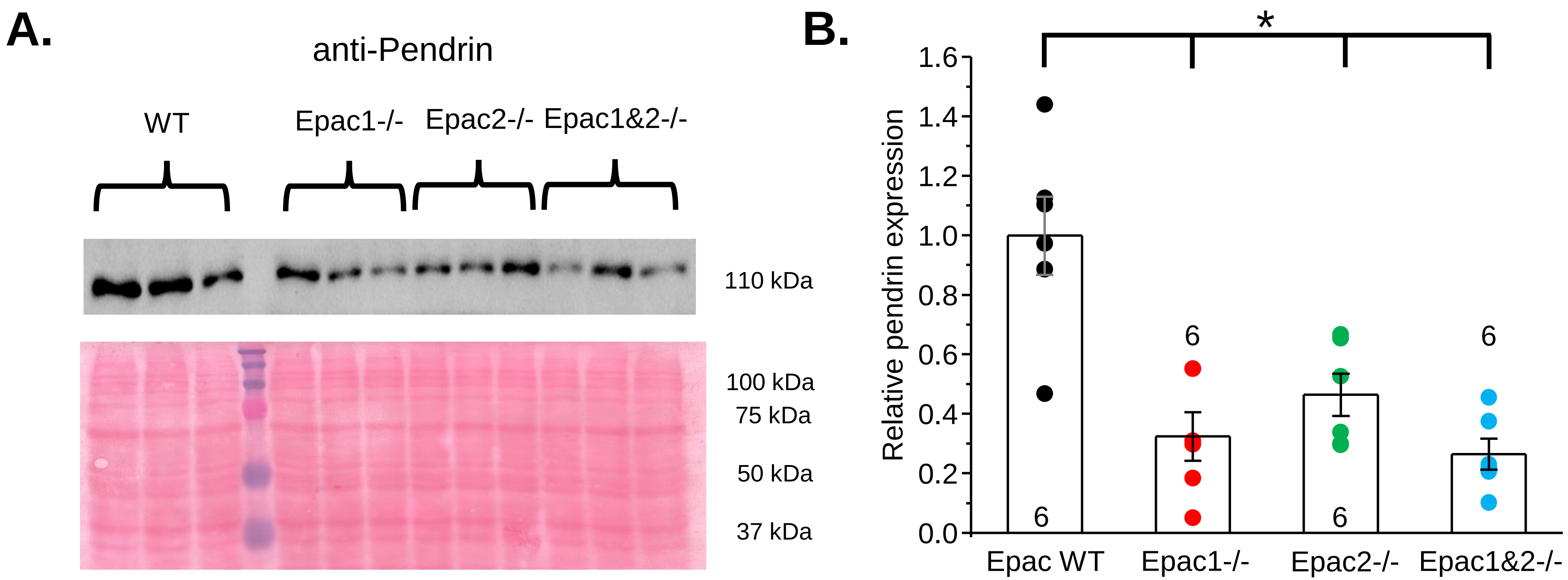

Supplemental Figure 6

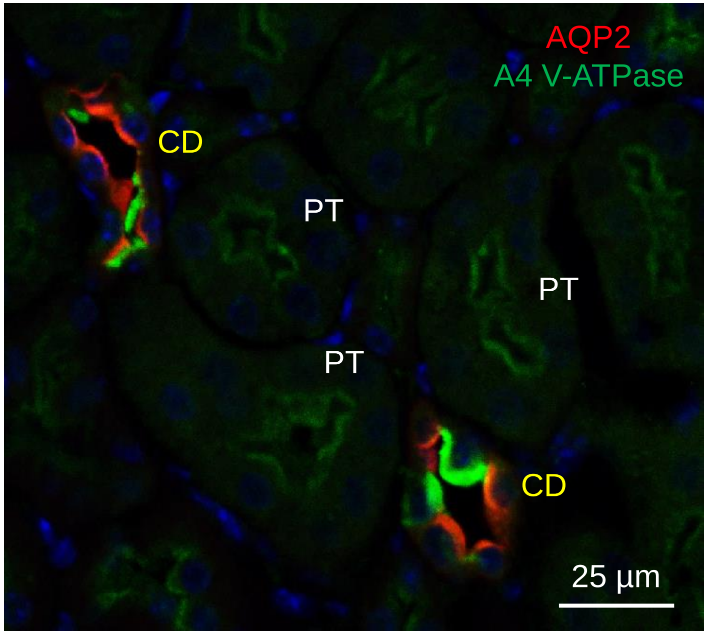

Supplemental Figure 7

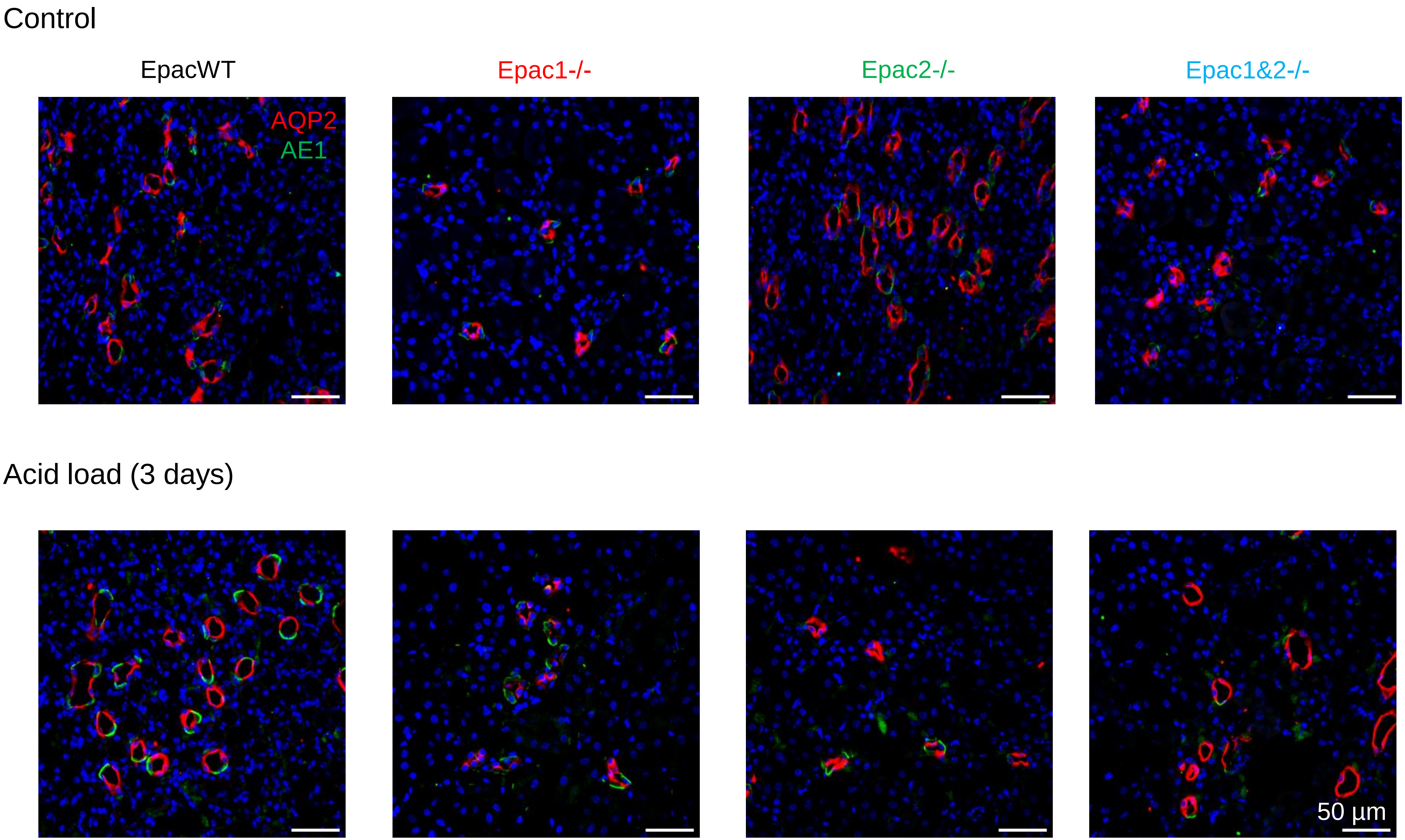

# Uncropped blots main figures

Figure 3

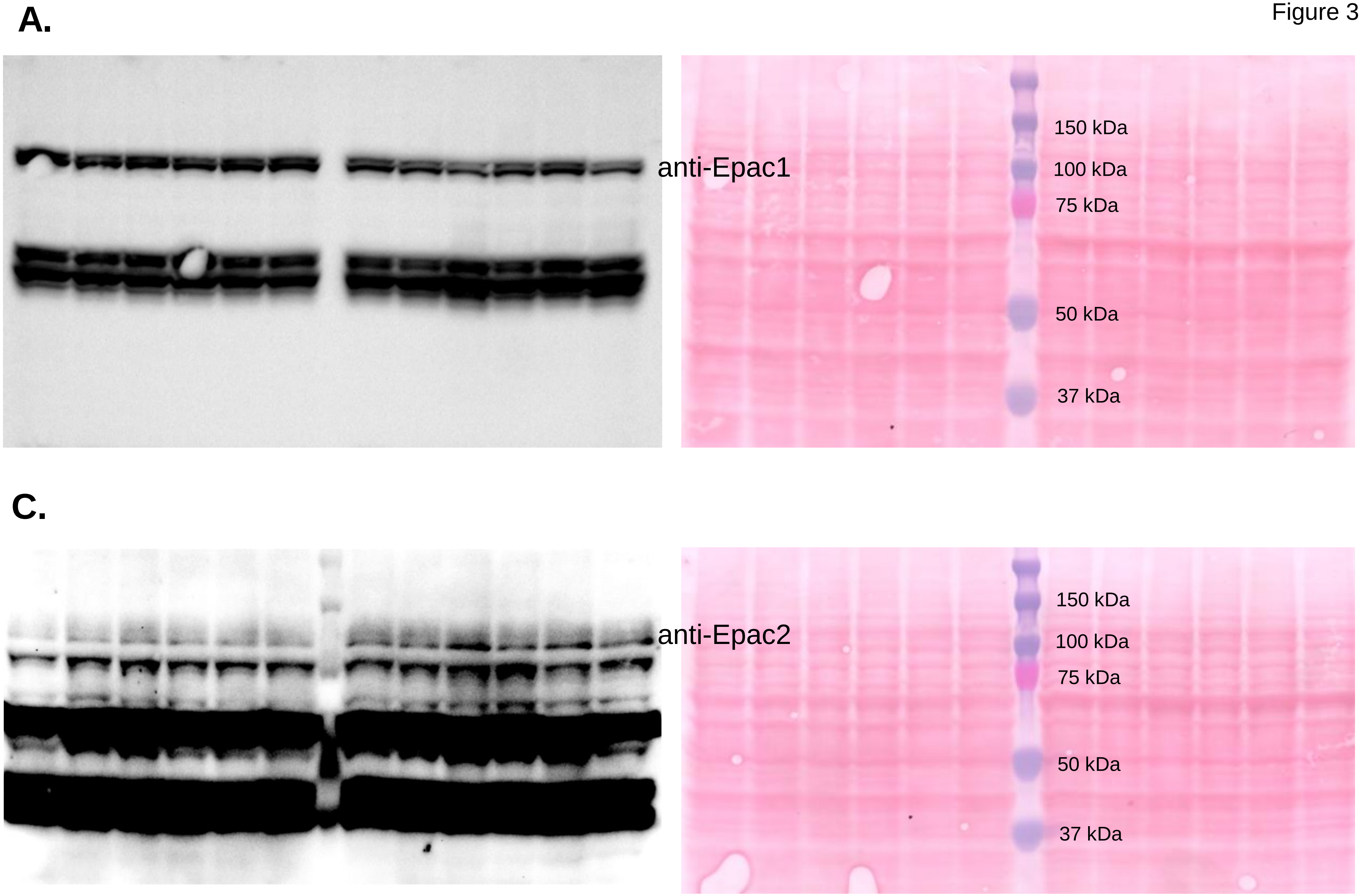
A.
anti-Epac1
150 kDa
100 kDa
75 kDa
50 kDa
37 kDa
C.
anti-Epac2
150 kDa
100 kDa
75 kDa
50 kDa
37 kDa

Figure 4

**A.**

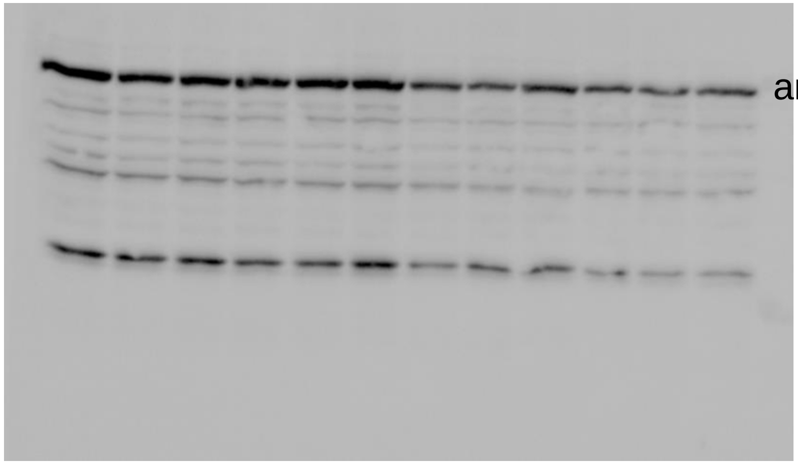
anti-NHE-3

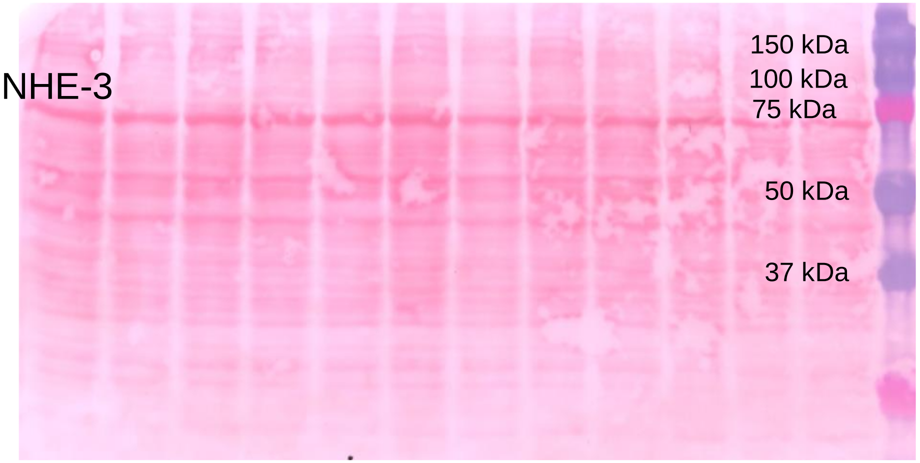
150 kDa
100 kDa
75 kDa
50 kDa
37 kDa

**C.**

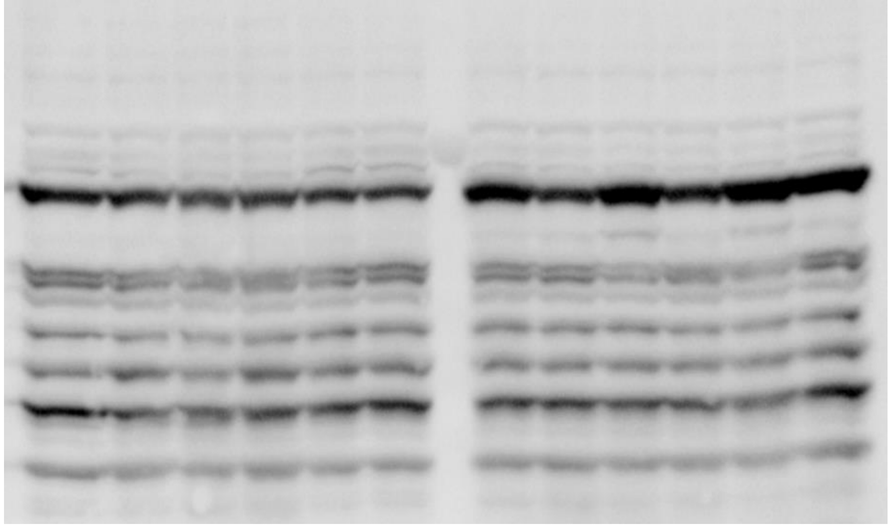
EpacWT
anti-NHE-3

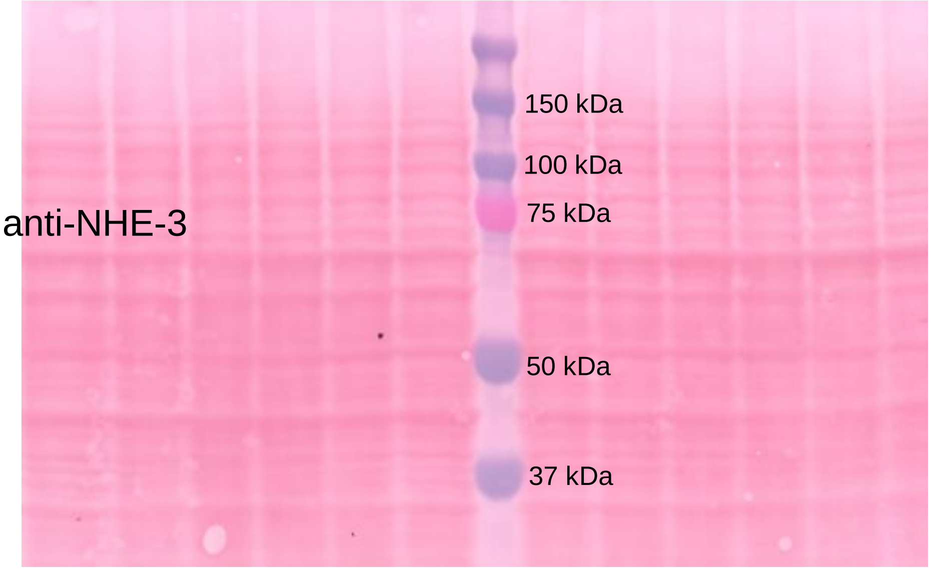
150 kDa
100 kDa
75 kDa
50 kDa
37 kDa

C.

Figure 4

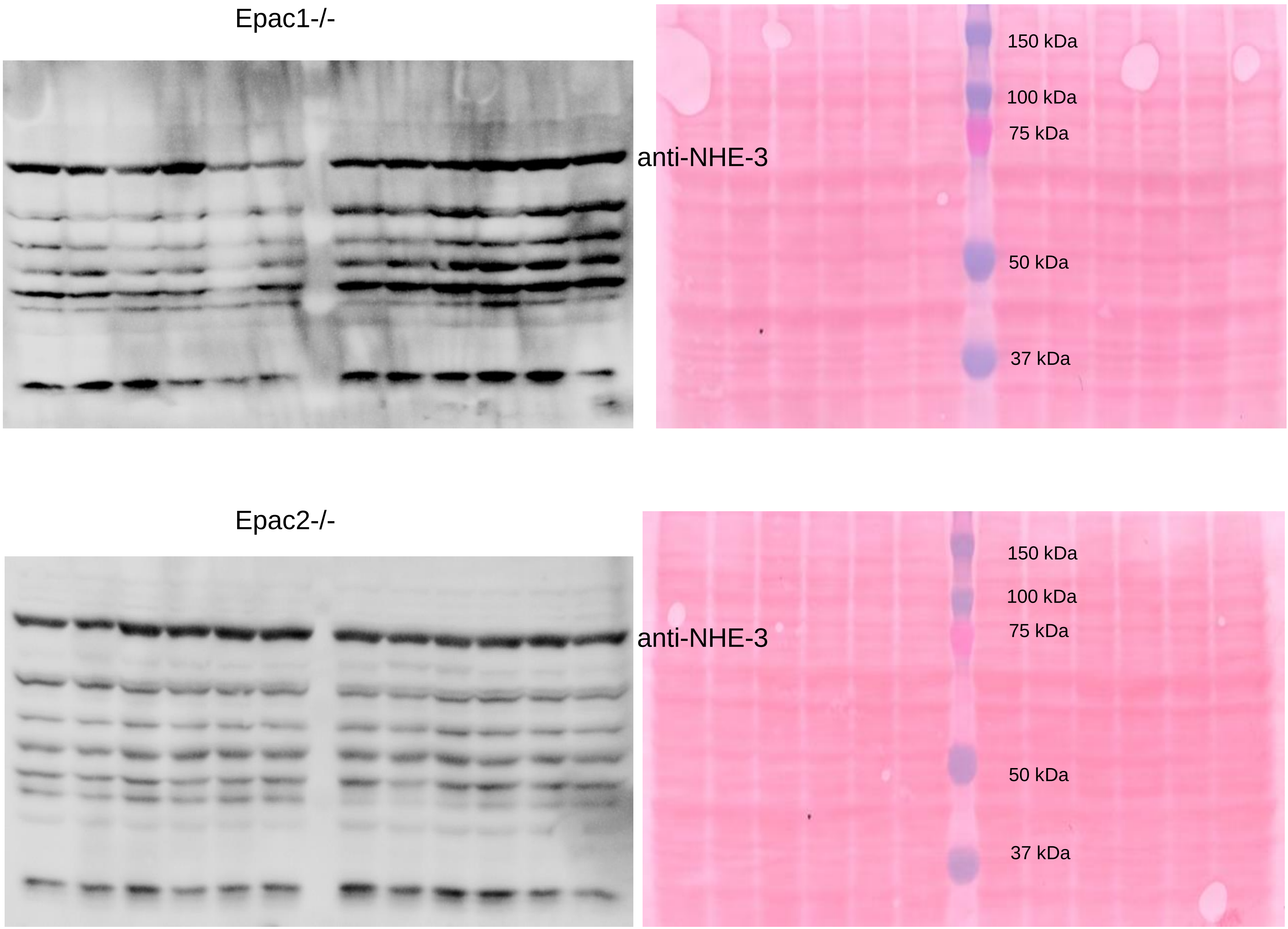
Epac1-/-
anti-NHE-3
150 kDa
100 kDa
75 kDa
50 kDa
37 kDa
Epac2-/-
anti-NHE-3
150 kDa
100 kDa
75 kDa
50 kDa
37 kDa

**C.**

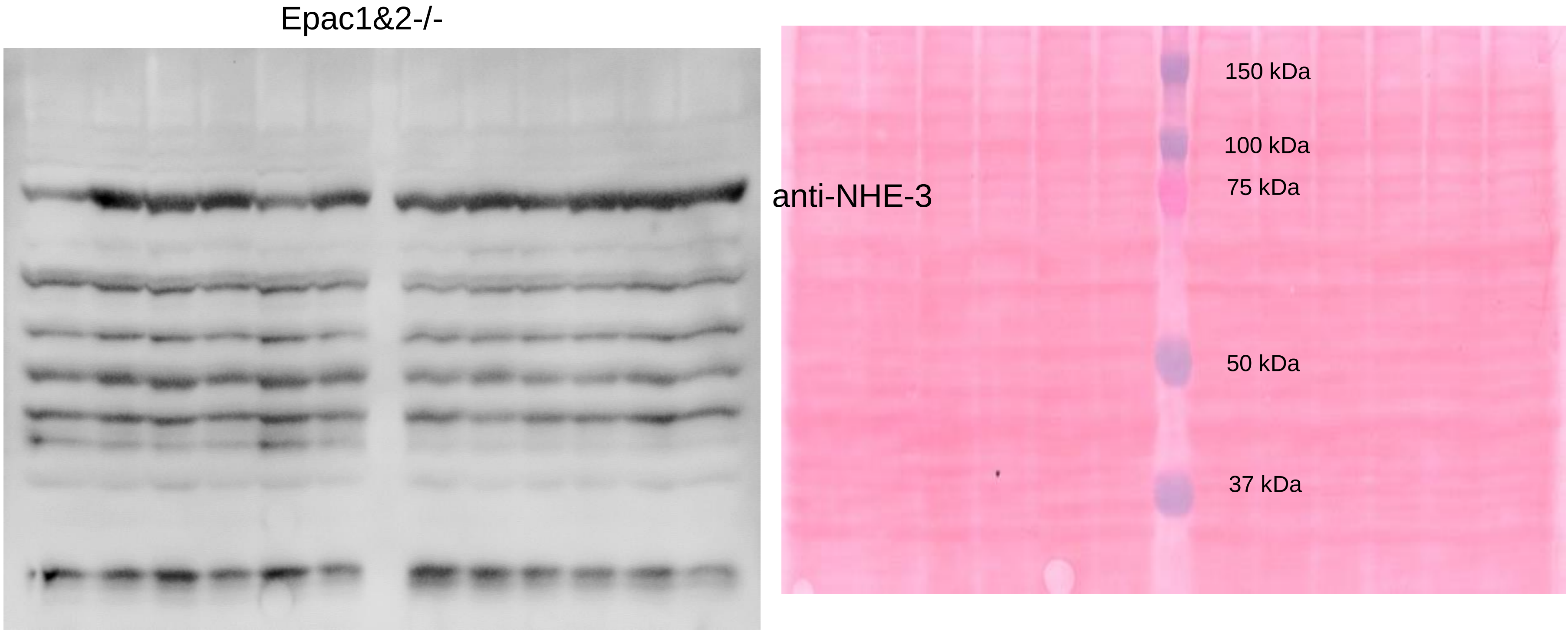

**B.**

Figure 9

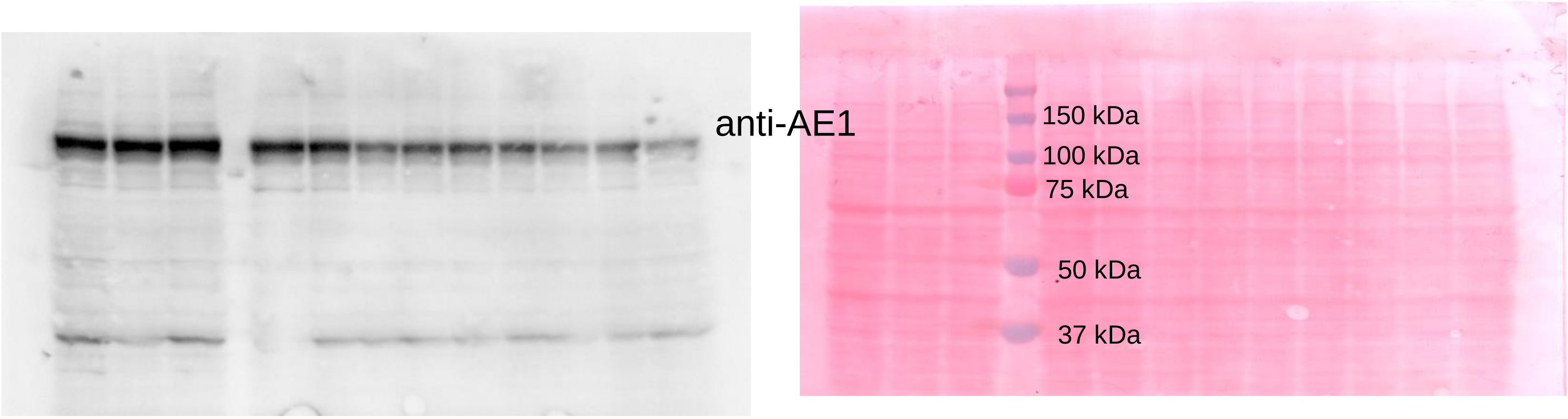

# Uncropped blots supplementary figures

Supplemental Figure 4

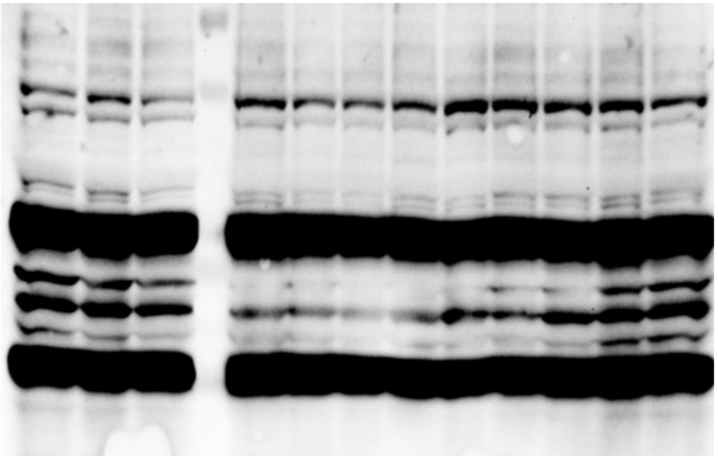

anti-NBCe1

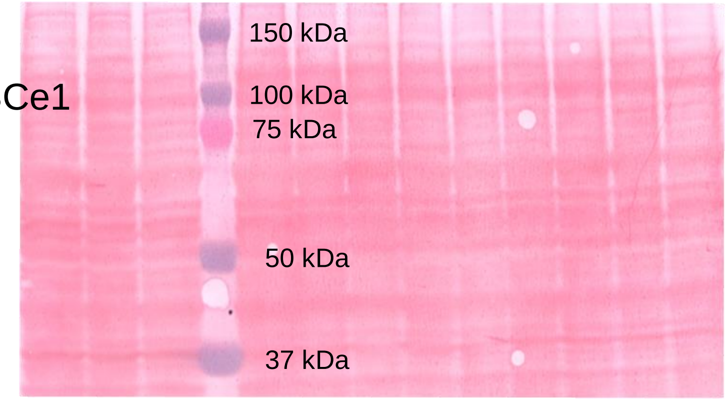

Supplemental Figure 5

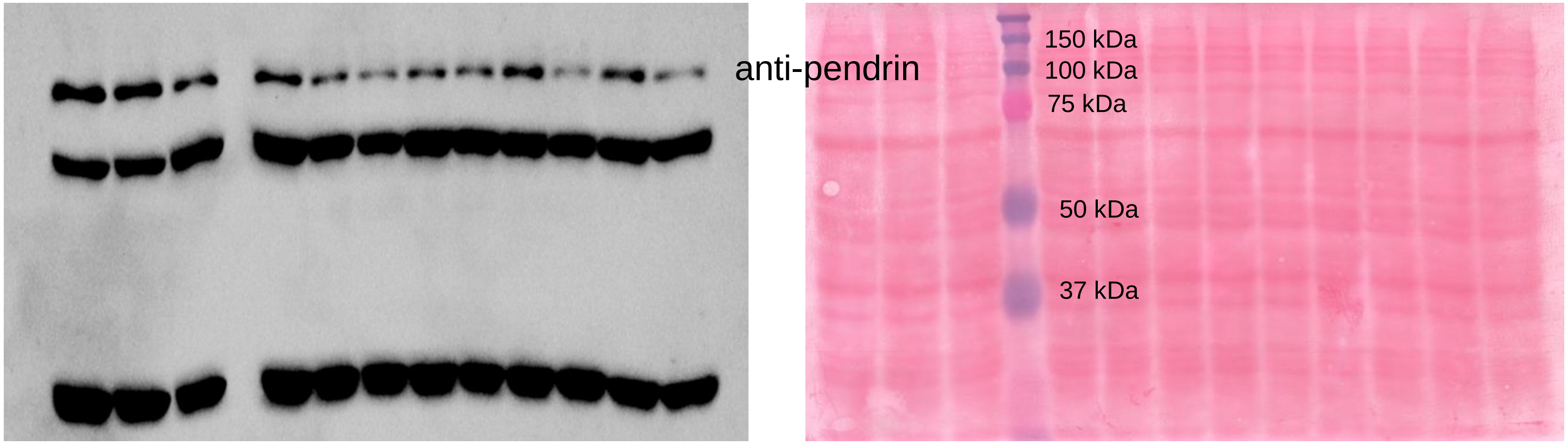

Supplement: zqaf048_Supplemental_Files [file zqaf048_supplemental_files.zip › Supplementary+ Uncropped Figures Revision Final.pdf]
