# Supplementary material for: Epac2 Deficiency Compromises Adaptation to Dietary Acidification by Decreasing H+ Transport in the Renal Nephron
Source: Function (Oxf). 2025 Oct 14;6(6):zqaf048. doi: 10.1093/function/zqaf048 (PMC12586993; doi:10.1093/function/zqaf048)
Supplement: zqaf048_Supplemental_Files [file zqaf048_supplemental_files.zip › Coverletter to Function.docx]

Dear Editor:

We are submitting the attached article by Pyrshev and colleagues entitled “**Epac2 deficiency compromises adaptation to dietary acidification by decreasing H^+^ transport in the renal nephron**” as a research article for consideration in the Function.

In this study, we uncover a novel physiologically relevant role of the exchange protein directly activated by cAMP (Epac) signaling cascade and more specifically Epac2 isoform in maintaining acid-base balance in response to dietary acidification. Using a broad spectrum of high throughput, genetical, biochemical, molecular biology tools in combination with advanced imaging techniques in native kidney proximal tubule and collecting ducts we demonstrate that deletion of Epac2 decreases the apical Na^+^/H^+^ exchange in the proximal tubule by reducing Na^+^/H^+^ exchanger, NHE-3 levels and targets the transporter to the base of brush border where it is less active. Second, it impedes H^+^ secretion by A-type intercalated cells of the collecting duct in part by decreasing expression of the AE1 anion exchanger. While Epac2-/- mice are able to hold physiological pH at the baseline at the expense of lower urinary pH, dietary acidification unmasks the deficient H^+^ secretion resulting in severe metabolic acidosis due to inability to further acidify urine. We further show that the regulation of systemic acid-base balance by Epac signaling cascade appears to be isoform-specific, since deletion of Epac1 has considerably weaker effect on NHE-3 levels and does not affect H^+^ secretion in the collecting duct. Epac1-/- mice have normal urinary pH at the baseline and are able to decrease it in response to dietary acidification exhibiting almost normal adaptation seen in EpacWT animals.

All authors have read and approved the submission of the manuscript; the manuscript has not been published and is not being considered for publication elsewhere, in whole or in part, in any language, except as an abstract.

We appreciate your consideration of our manuscript. We look forward to hearing from you.

Sincerely,

Oleh Pochynyuk, PhD, FAHA

Professor

Department of Integrative Biology and Pharmacology

University of Texas Health Science Center at Houston
